# Integrative Genomic and Transcriptomic Analysis Identified Candidate Genes Implicated in the Pathogenesis of Hepatosplenic T-Cell Lymphoma

**DOI:** 10.1371/journal.pone.0102977

**Published:** 2014-07-24

**Authors:** Julio Finalet Ferreiro, Leila Rouhigharabaei, Helena Urbankova, Jo-Anne van der Krogt, Lucienne Michaux, Shashirekha Shetty, Laszlo Krenacs, Thomas Tousseyn, Pascale De Paepe, Anne Uyttebroeck, Gregor Verhoef, Tom Taghon, Peter Vandenberghe, Jan Cools, Iwona Wlodarska

**Affiliations:** 1 Center for Human Genetics, KU Leuven, Leuven, Belgium; 2 Molecular Pathology, Cleveland Clinic, Cleveland, Ohio, United States of America; 3 Laboratory of Tumor Pathology and Molecular Diagnostics, University of Szeged, Szeged, Hungary; 4 Translational Cell and Tissue Research KU Leuven, Department of Pathology UZ Leuven, Leuven, Belgium; 5 Department of Pathology, AZ St Jan AV, Brugge, Belgium; 6 Department of Pediatrics, UZ Leuven, Leuven, Belgium; 7 Department of Hematology, UZ Leuven, Leuven, Belgium; 8 Department of Clinical Chemistry, Microbiology and Immunology, Ghent University Hospital, Ghent University, Ghent, Belgium; 9 Center for the Biology of Disease, VIB, Leuven, Belgium; Deutsches Krebsforschungszentrum, Germany

## Abstract

Hepatosplenic T-cell lymphoma (HSTL) is an aggressive lymphoma cytogenetically characterized by isochromosome 7q [i(7)(q10)], of which the molecular consequences remain unknown. We report here results of an integrative genomic and transcriptomic (expression microarray and RNA-sequencing) study of six i(7)(q10)-positive HSTL cases, including HSTL-derived cell line (DERL-2), and three cases with ring 7 [r(7)], the recently identified rare variant aberration. Using high resolution array CGH, we profiled all cases and mapped the common deleted region (CDR) at 7p22.1p14.1 (34.88 Mb; 3506316-38406226 bp) and the common gained region (CGR) at 7q22.11q31.1 (38.77 Mb; 86259620–124892276 bp). Interestingly, CDR spans a smaller region of 13 Mb (86259620–99271246 bp) constantly amplified in cases with r(7). In addition, we found that *TCRG* (7p14.1) and *TCRB* (7q32) are involved in formation of r(7), which seems to be a byproduct of illegitimate somatic rearrangement of both loci. Further transcriptomic analysis has not identified any CDR-related candidate tumor suppressor gene. Instead, loss of 7p22.1p14.1 correlated with an enhanced expression of *CHN2* (7p14.1) and the encoded β2-chimerin. Gain and amplification of 7q22.11q31.1 are associated with an increased expression of several genes postulated to be implicated in cancer, including *RUNDC3B*, *PPP1R9A* and *ABCB1*, a known multidrug resistance gene. RNA-sequencing did not identify any disease-defining mutation or gene fusion. Thus, chromosome 7 imbalances remain the only driver events detected in this tumor. We hypothesize that the Δ7p22.1p14.1-associated enhanced expression of *CHN2*/β2-chimerin leads to downmodulation of the NFAT pathway and a proliferative response, while upregulation of the CGR-related genes provides growth advantage for neoplastic δγT-cells and underlies their intrinsic chemoresistance. Finally, our study confirms the previously described gene expression profile of HSTL and identifies a set of 24 genes, including three located on chromosome 7 (*CHN2, ABCB1* and *PPP1R9A*), distinguishing HSTL from other malignancies.

## Introduction

Hepatosplenic T-cell lymphoma (HSTL) is a rare and clinically aggressive subtype of peripheral T-cell lymphoma (PTCL) [1], recognized as a distinct clinico-pathological entity in the 2008 WHO classification [2]. Patients, predominantly young men, usually present with isolated hepatosplenomegaly and thrombocytopenia. Histologically, they show sinusoidal involvement of bone marrow, liver and spleen. HSTL is derived from the γδ (occasionally αβ [3]–[5]) cytotoxic memory T-cells responsible for innate immunity. The disease shows a fulminant clinical course, therapy resistance and poor prognosis. The median survival of patients with HSTL is usually shorter than two years [6]. Cytogenetically, isochromosome 7q [i(7)(q10)] is a hallmark of HSTL [7]–[10], although sporadic cases with a ring chromosome 7 [r(7)] [11]–[13] or translocation involving chromosome 7 [14] have been published. The most common accompanying karyotype alteration is trisomy 8 [9]. Thus far, the functional and molecular genetic consequences of i(7)(q10) in HSTL remain largely unknown. As development of i(7)(q10) results in loss of one copy of the short arm of chromosome 7 (7p) and gain of the long arm of chromosome 7 (7q), neoplastic cells presumably suffer from an aberrant gene dosage effect. Some or all of these imbalances may represent the key event driving the development of HSTL. As HSTL tends to gain extra copies of i(7)(q10) [10], [15] or selectively amplify 7q sequences [11]–[13], overrepresentation of 7q seems to have an important impact on the pathogenesis of this lymphoma. Combined gene expression profiling (GEP) and array-based comparative genomic hybridization (aCGH) of several HSTL cases recently reported by Travert *et al*. [5] showed downregulation of 7p genes, particularly *CYCS, IKZF1, HUS1* and *CBX3*, and upregulation of 7q genes, including the putative oncogene *PTPN12*. To gain further insight into the molecular pathogenesis of HSTL, we determined genomic profiles of six i(7)(q10)-positive HSTL cases, including DER-L2 cell line, and three cases with r(7), and significantly narrowed down the common deleted region on 7p and the common gained region on 7q. Subsequent transcriptomic studies using global microarray expression profiling and RNA-sequencing led to identification of candidate genes implicated in the pathogenesis of HSTL.

## Materials and Methods

### Patients

Six HSTL cases were collected from files of the Center for Human Genetics, KU Leuven, Leuven, Belgium. Two cases were provided by L. Krenacs (Laboratory of Tumor Pathology and Molecular Diagnostics, Szeged, Hungary) and S. Shetty (Department of Medical Genetics, Alberta Children's Hospital, Calgary, Canada). Diagnosis of HSTL was based on histopathology and immunophenotype, according to the WHO criteria [2]. The clinical, pathological and immunophenotypic features of the patients were reviewed. The study was approved by the institutional review board “Commissie Medische Ethiek” of the University Hospital. For this retrospective study the “Commissie Medische Ethiek” waived the need for written informed consent from the participants.

### Cytogenetics and fluorescence *in situ* hybridization

R- and G-banding chromosomal analysis and fluorescence *in situ* hybridization (FISH) analysis followed standard procedures. Probes used for FISH analysis are listed in Table S1. Non-commercial probes were labeled with SpectrumOrange- and SpectrumGreen-d-UTP (Abbott Molecular, Ottigne, Belgium) using random priming. FISH experiments were evaluated using an Axioplan 2 fluorescence microscope equipped with a charge-coupled device Axiophot 2 camera (Carl Zeiss Microscopy, Jena, Germany) and a MetaSystems Isis imaging system (MetaSystems, Altlussheim, Germany). Two to 10 abnormal metaphases and/or 200 interphase cells were evaluated in each FISH experiment.

### High resolution array CGH

Total genomic DNA was isolated from fresh frozen lymphoma samples or cytogenetic pellet (Table 1; case 2) using standard procedures. Genomic profiling, following the manufacturer's protocols, was performed using the Agilent 244k (www.agilent.com) (5 cases) and the Affymetrix CytoScan HD arrays (www.affymetrix.com) (4 cases). Array CGH data are available at GEO (Accession number: GSE57944).

**Table 1:** Relevant clinical and genetic data.

Case	Sex/Age	Previous medical history	Histologically proven sites of involve-ment	Treatment	Outcome (months)	Cytogenetics		aCGH platform	PHF14 seq	GEP Affy 2.0	RNAseq	WB
						Sample/status	Karyotype^b^					
**1**	M/26		S	splenectomy, allo BMTx	CR, alive (80)	S/D	46–48,XY,r(7),inc[2]	Agilent 244k	done	done		
**2** a	M/7	possible IgA nephropathy	S, L, BM	splenectomy, combined CT (POG 9404 induction protocol)	CR (12), lost to FU	BM/D	47,XY,r(7)(p22q36),+8[15]	Agilent 244k				
**3** a	F/62	ITP	S, L, BM	splenectomy, combined CT	DOD (2)	BM/D	47,XX,r(7),+8,der(19)t(?;19)(?;p13)[10]	Agilent 244k				
**4** a	M/33	Budd-Chiari syndrome, liver Tx	S, L, BM	splenectomy	DOD (2)	S/D	45–46,X,-Y,-4,der(7)add(7)(p22)add(7)(q32), i(7)(q10),+i(7)(q10)[2],der(8)t(1;8)(q21; p23),-22,+mar1,+mar2[cp11].aCGH+8	Affymetrix CytoScan HD		done	done	done
**5**	M/52	Crohn's disease	S	splenectomy, combined CT, MAB	DOD (11.5)	S/D	46–47,XY,add(4)(p16),i(7)(q10),+8[4],-[20], +mar[2][cp6]	Agilent 244k	done	done	done	done
**6**	M/50		S, BM	splenectomy, combined CT	DOD (25)	S/D	43–45,X,-Y,i(7)(q10)[cp7].aCGH+8	Affymetrix CytoScan HD	done			
**7**	M/18	kidney Tx for dysplasia	S, Pe, BM	splenectomy, combined CT	DOD (8.5)	S/D	40–48,XY,+X [3],-5[4],i(7)(q10),+8[5],+10[2],add(11)(q22)[10],inc[cp12].aCGH+8	Affymetrix CytoScan HD		done	done	done
**8**	F/55		S, BM	splenectomy, combined CT	DOD (21)	BM/P	46–47,XX,i(7)(q10),+i(7)(q10)[4],+8,-10, add(15)(q26),add(22)(q13)[cp12]	Affymetrix CytoScan HD				
**9** **DERL-2**							46,XY,add(5)(q?),i(7)(q10),-10[5][cp15]^c^. aCGH+8	Agilent 244k	done		done	done

a , previously published cases[10],[11], [13].

b , karyotypes were described according to recommendations of ISCN (2013)[84] ^c^, karyotype according to Di Noto *et al*.(2001)[22].

abbreviations: ITP, idiopathic thrombocytopenic purpura; POG, Pediatric Oncology Group; MAB, Monoclonal Antibodies; Tx, transplantation; S, spleen; L, liver; BM, bone marrow; Pe, peritoneum; allo, allogeneic; CT, chemotherapy; FU, follow-up; D, diagnosis; P, progression; WB, Western blotting.

### Data analysis and visualization software

Downstream data analysis of the genomic profiling results was performed using the software ArrayStudio, version 6.2 (www.omicsoft.com). Unless otherwise specified, this software was also used for various analysis performed on the expression data retrieved from microarray and RNAseq technologies described below.

### PHF14 sequencing

Mutation analysis of *PHF14* was performed on total genomic DNA from five index cases (Table 1) and four control PTCL cases without chromosome 7 abnormalities. PCR amplification and sequence analysis of genomic sequences spanning full exons of *PHF14* were performed using Sanger sequencing primers (Table S2) and conventional sequencing method.

### 454 sequencing

Custom designed Nimblegen sequence capture 385k Version 2.0 Arrays (Roche Applied Science, Mannheim, Germany) targeting sequences at 7p21.3/10106629-11176525 (hg18) were produced. Preparation of shot-gun DNA sequencing libraries and capturing of the target region was performed according to the manufacturer's instructions. Captured DNA was pyrosequenced on a GS FLX instrument (Roche Applied Science, Mannheim, Germany) according to the manufacturer's instructions.

### Microarray gene expression analysis

Total RNA extraction from four frozen lymphoma samples (Table 1) and three nonmalignant spleens was performed using TRIzol LS Reagent (Life Technologies Europe B.V., Ghent, Belgium). For gene expression profiling, the Affymetrix platform HG-U133 Plus 2.0 was used. To increase the statistical significance of the study, data from 13 previously published HSTL cases, various T-cell malignancies [25 cases of PTCL (peripheral T-cell lymphoma), 10 cases of NK/TCL (Natural Killer/T-cell lymphoma), 21 cases of AITCL (angioimmunoblastic T-cell lymphoma) and nonmalignant samples (6 spleens, 26 samples of T-cells, including activated γδT-cells] were retrieved from public sources (GEO and ArrayExpressed)) (Table S3). The raw data of all cases (CEL files) were normalized together using the GeneChip-Robust Multiarray Averaging (GC-RMA) algorithm. Principal component analysis (PCA), hierarchical clustering and a special application of Lewi's spectral mapping [16] to microarrays (Spectral Map Analysis, SMA) (www.vetstat.ugent.be/workshop/Nairobi2004/Bijnens/Bijnens2004.pdf) were used to detect relationship in the data and to identify outliers. To find differentially expressed genes, the General Linear Model (GLM) was used for inference analysis. The resulting Fold Change (FC) and False Discovery Rate (FDR) (using the Benjamini–Hochberg procedure, FDR_BH) were used to set differential expression cut-offs. The cut offs values for FC ranged from an absolute value (Abs(FC)) of 2.0 (Abs(FC) ≥2.0) to 3.5 (Abs(FC)≥3.5). The maximum FDR used as a cut off was 0.1 (FDR ≤0.1) and the minimum was 0.05 (FDR ≤0.05). The microarray data of four index cases and three nonmalignant spleens were deposited in GEO (Accession number: GSE57944).

### Library preparation for paired-end RNA-sequencing and processing of RNAseq reads

Four samples of HSTL (Table 1) and one nonmalignant spleen sample were subjected to RNA-sequencing. The Illumina standard kit (Illumina TruSeq RNA Sample Preparation Kit, San Diego, CA, USA) was used for the mRNAseq sample preparation according to the manufacturer's protocol. Briefly, 1 µg of total RNA was used for polyA mRNA selection using poly-T oligo-attached magnetic beads, followed by thermal mRNA fragmentation. Using reverse transcriptase (Superscript II, Life Technologies Europe B.V.e, Ghent, Belgium) and random primers, cDNA was synthesized and subsequently double stranded, end-repaired (End Repair Mix) and ligated to the Illumina RNA Indexes Adaptor. The libraries were purified after enrichment using 15 cycles of PCR. The insert sizes of the libraries were checked by Agilent Technologies 2100 Bioanalyzer.

### Processing of Illumina RNA-sequencing reads

Prepared libraries were sequenced using HiSeq 2000 (Illumina, San Diego, CA, USA) operated in paired-end 2×100 bp mode. Reads were quality-filtered using a standard Illumina process.

### RNAseq bioinformatics analysis

For further analysis, we used additional RNAseq data of three cases of PTCL, five cases of T-ALL, the Jurkat T-cell line and one nonmalignant thymus available in our institution. The fastq files of all samples were mapped to the reference human genome (assembly GRCh37.68). The mapping was performed using OSA [17] with the default parameters allowing detection of insertions and deletions (indels). The mapped reads were used to calculate read counts and FPKM (Fragment Per Kilobase of exon model per Million of mapped read) per gene. The DESeq algorithm [18] was applied to identify differentially expressed genes. Prediction of SNV followed the previously described approach [19] filtering out the variants found in normal spleen. The detection of indels was done independently using ArrayStudio. Fusion transcript discovery was performed using deFuse v.0.5.0 [20] with default parameters and a fusion detection algorithm provided by ArrayStudio [17], [21]. Fusions with less than 8 spanning reads and less than 5 split reads were filtered out as well as those observed in adjacent genes.

### Gene signature analysis

To find a gene signature of HSTL, at first we ran 10 different inference analyses (using both microarray and RNAseq data) comparing HSTL with PTCL-NOS, NK/TCL, AITCL, T-ALL, nonsorted normal T-cells, sorted activated γδT-cells, nonmalignant spleen and thymus. After selecting the differentially expressed genes in every comparison, the results (FC of the gene per comparison) were merged in a table. Then, we selected genes with a consistent expression pattern (either up- or downregulated) across the different comparisons and used these genes for unsupervised hierarchical clustering analyses. The resulting dendograms and heatmaps were visually inspected and the genes which were not essential to keep the integrity of the HSTL samples cluster were removed. This process was repeated until a minimal number of genes was found, which keep the cluster formed by the HSTL samples intact.

### Biological pathways analysis

To find significant enriched pathways and biological functions in HSTL, we uploaded the result of the inference analyses into the “Ingenuity Pathway Analysis” application (IPA, www.ingenuity.com). From the three confidence levels provided by the system, we used “Experimentally observed” and “Highly predicted” data. For details see: http://ingenuity.force.com/ipa/articles/Feature_Description/Canonical-Pathways-for-a-Dataset

### QRT-PCR analysis

Quantitative RT-PCR was performed with the LightCycler 480 SYBR Green I master mix (Roche Diagnostics Belgium, Vilvoorde, Belgium) and the results were analyzed using the comparative dCt method. The analysis was done using 3 replicates and the two most similar values were used to calculate the mean. Primer sequences are shown in Table S2.

### Western blot analysis

Sections from lymphoma frozen tissues and pellets of cultured DERL-2 cells were lysed and processed for Western blotting according to standard procedure using antibodies against β2-chimerin (2E3; Rat mAb nr. 4728, Cell Signaling Technology, Danvers, Massachusetts, USA) and β-actin (Sigma Aldrich, St. Louis, MO, USA). Protein detection was performed with Image Quant Las4000. Densitometric analysis of protein blots was performed using the ImageJ (1.45) software from the National Institutes of Health (http://rsb.info.nih.gov/ij/).

### Immunohistochemistry

Expression of ABCB1 (MDR1) was analyzed by IHC with monoclonal MDR1 antibody (MAB4120, clone JSB-1, Millipore, Overijse, Belgium) used at dilution 1∶200 with a high pretreatment. Results were visualized using the OptiView DAB IHC Detection Kit (Ventana, Oro Valley, Tucson, Arizona). Image acquisition was done through a Leica microscope at 200× and 100× magnification. Images were assembled using Adobe Photoshop CS5.

## Results

### Clinical characteristics of HSTL patients

The relevant clinical features of eight reported HSTL cases are shown in Table 1. There were six male and two female patients in age ranging from seven to 62 years (average 38.7). The cases displayed the common clinical, morphological and immunophenotypic features of HSTL, including γδT-cell origin [2]. Histologically, all patients showed spleen involvement. Liver and/or bone marrow (BM) involvement were histologically proven in three and six patients, respectively. Four patients (50%) had either a previous autoimmune disorder (i.e. idiopathic thrombocytopenic purpura, Crohn's disease) or underwent immunosuppressive treatment after solid organ transplantation. All patients underwent splenectomy, which was followed by allogenic BM transplantation (alloBMTX) (one case) or combined chemotherapy (CT) (six cases). One patient died two months after diagnosis, six patients treated with combined CT survived 2-25 months (average 11.7 months) and one patient was lost for follow-up after 12 months of complete remission (CR). Notably, patient 1 who received alloBMTX is alive and remaining in CR (80 months).

### Molecular cytogenetic analysis

All eight patients and the included HSTL-derived DERL-2 cell line [22] displayed abnormal karyotypes with either i(7)(q10) (six cases) or r(7) (three cases) (Table 1). Of note, case 4 revealed additional aberrations of the other chromosome 7 and a subclonal duplication of i(7)(q10), which was also detected in case 8. Trisomy 8 was identified in five patients. The cases were further subjected to a high resolution aCGH analysis which detected genomic imbalances in all of them (Table S4).

**Figure 1:**
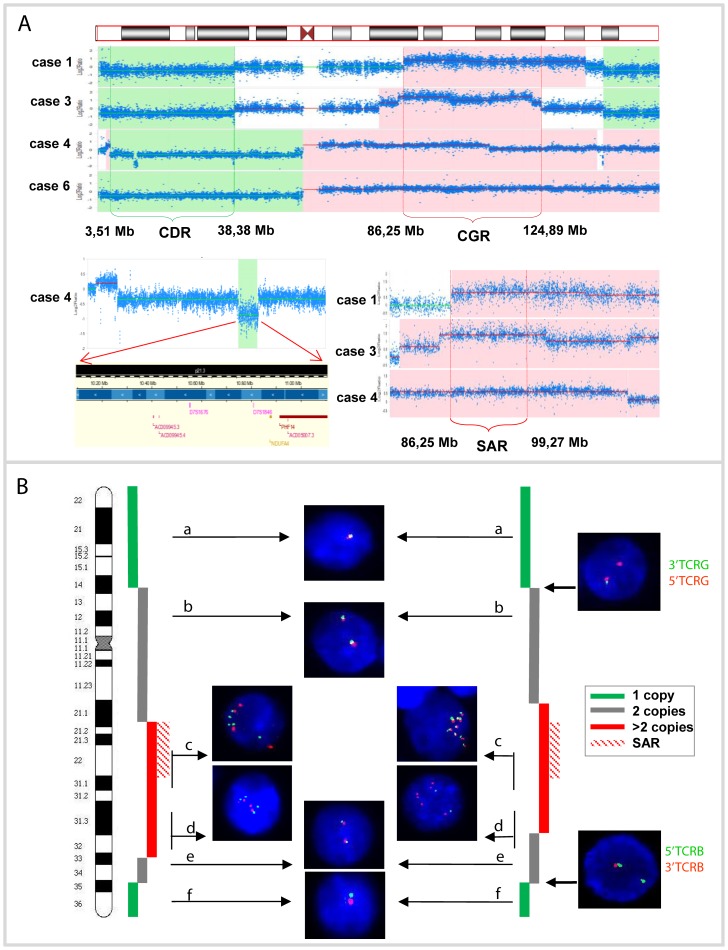
Examples of aCGH and FISH analysis. A) upper panel: genomic profile of chromosome 7 of four index cases with the indicated common deleted region (CDR) on 7p and common gained region (CGR) on 7q; lower left panel: gene content (Hg19) of the biallelically deleted 7p21 interval in case 4; lower right panel: zoomed CDR with the indicated selectively amplified region (SAR). B) Examples of interphase FISH validation of aCGH results performed in cases 1 (left panel) and 2 (right panel) with r(7). Applied probes: (a) RP11-99J06-SG/RP11-735O20-SO, (b) RP13-11C11-SG/RP11-807G04-SO, (c) RP11-513N08-SG/RP11-514N09-SO, (d) RP11-379L24-SO/RP11-16K22-SG,(e) RP11-269N18-SG/RP5-894A10-SO, (f) RP4-548K24-SG/RP11-135F23-SO.

**Figure 2:**
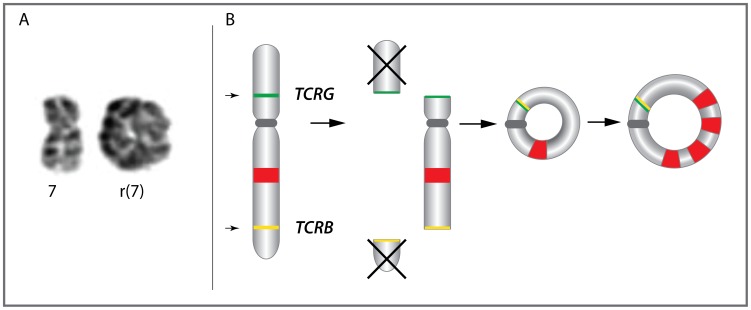
Mechanism underlying formation of r(7) in HSTL. A) Partial karyotype showing r(7). B) Proposed model of the r(7) formation. Illegitimate somatic rearrangement of *TCRB* and *TCRG* in δγT-cells leads to the aberrant *TCRB-TCRG* lesion and consequently to the formation of r(7) and loss of the terminal 7p and 7q regions, respectively. This process is followed by a subsequent gain/amplification of 7q sequences (shown in red).

Chromosome 7 profiles of cases 1 and 3 with r(7) were very similar. They were characterized by terminal losses of 7p/7q regions, with respective breakpoints at 7p14.1/*TCRG* (38406226 bp) and 7q32/*TCRB* (142502221 bp), and gain of 7q, which encompasses 7q21.12q33 (86259620–137506193 bp) in case 1 and 7q21.11q31.33 (79158260–124892276 bp) in case 3 (Figure 1A, upper panel). The profile of case 2 was less pronounced (<20% of abnormal cells), although loss of 7p22.3p22.1 and gain of 7q21.1q32.1 were evident. Five cases with i(7)(q10) revealed loss of the entire 7p and gain of 7q, as expected. Case 4 displayed complex imbalances, including duplication of the terminal 7p22.2p22.2 (2347596–3506315 bp) region, monoallelic loss of 7p22.1p11 (3506316–57883626 bp) and a biallelic microdeletion at 7p21.3 (10165499–11213632 bp) (Figure 1A, left lower panel). The long arm of chromosome 7 showed three copies of the 7q11q31.1 (61.831.840–110403720 bp) region and two copies of 7q31.1q36 (110413108–159118566 bp). Based on the aCGH data, we defined the common deleted region (CDR) at 7p22.1p14.1 (3506316–38406226 bp) (34.89 Mb) and the common gained region (CGR) at 7q21.22q31.1/(86259620–124892276 Mb) (38.78 Mb) (Figure 1A, upper panel). Cases 1 and 3 displayed a smaller amplified region (SAR)of 13 Mb (86259620–99271246 bp) at 7q21.22q22.1 (Figure 1A, right lower panel). To validate aCGH data, we analyzed two available cases with r(7) (cases 1 and 2) by interphase FISH with the selected 7p/7q BAC clones (Table S1). As illustrated in Figure 1B, FISH confirmed the localization of terminal 7p/7q breakpoints within *TCRG* and *TCRB*, respectively, and the associated loss of terminal sequences flanking both loci, suggesting their involvement in formation of r(7) (Figure 2). FISH also evidenced a different size of the gained 7q region and the level of 7q gain. For example, three SAR-related probes showed 4–5 signals in case 1 and 6–9 signals in case 2 (Figure 1B).

Other recurrent aCGH imbalances detected in at least two cases include a trisomy 8 (7 cases), duplication of 1q (3 cases; common gain of 1q31q34), loss of 4p (3 cases; common loss of 4p16.3p16.3), loss of 10p (2 cases; common loss of 10p14p13/10p12.2p11.22) and duplication of 17q (2 cases; common gain of 17q21.33q25.3) (Table S4). Of note, trisomy 8 was not detected in case 2 (low proportion of tumor cells) but it was identified in three additional cases (no. 4, 6 and 9). Altogether, cytogenetics and aCGH detected trisomy 8 in eight out of nine (88.8%) studied cases.

### Sequencing of the biallelically deleted 7p21 region

The 7p21.3 region biallelically deleted in case 4 harbors two protein-coding genes, *NDUFA4* and *PHF14*, a candidate tumor suppressor gene (TSG) [23]–[25]. To examine the mutational status of *PHF14* on the nondeleted 7p allele in other index cases, we sequenced the gene in cases 1, 5, 6, and 9 (DERL-2). No mutation, however, was identified. In the next step, the entire 10165499–11213632 bp region was sequenced using the 454 technology combined with region capturing. This analysis detected neither recurrent intergenic nor intragenic mutations in the four samples analyzed.

### Quality of the RNAseq data

The average number of reads obtained was 98.7 million and the average percentage of uniquely mapped reads was 86.3%. No 3′ bias was detected. The full alignment report is in Table S5. The raw RNAseq data (fastq files) of all HSTL and PTCL-NOS cases analyzed plus the normal spleen are available at GEO (Accession number: GSE57944).

### Gene expression and pathway analysis

Transcriptome of HSTL was studied using expression microarray (MA) [cases 1, 4, 5, 7 plus 100 publicly available samples, including HSTL (n = 13), various T/NK-cell malignancies (n = 54) and normal T-cell controls (n = 33); Table S3] and RNAseq approach [cases 4, 5, 7, DERL-2 plus 10 control samples including, PTCL-NOS (n = 3), T-ALL (n = 5), nonmalignant spleen (n = 1) and thymus (n = 1)] (see details in Materials and Methods). Given that the MA dataset included samples from five different laboratories, at first we performed data structure analysis including Spectral Map Analysis (SMA), hierarchical clustering and Principal Component Analysis (PCA). SMA showed that the MA samples selected for further bioinformatic analysis cluster according to their classification and there was no bias by their laboratory origin (Figure S1 A–B). PCA on the RNAseq expression data discriminated HSTL from other analyzed samples except for the nonmalignant thymus (Figure S2).

**Figure 3:**
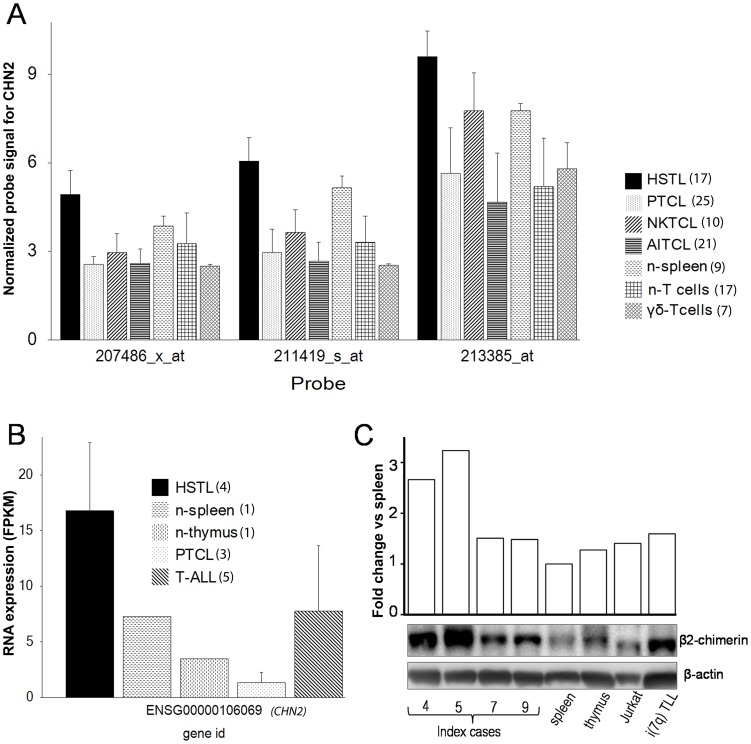
Expression of CHN2/β2-chimerin. A) Normalized values of all *CHN2* probes in the used U133 array for the analyzed malignancies and normal controls. The observed expression differences were statistically significant, with FDR_BH <0.05 (Table S6). B) Expression values of *CHN2* using the RNAseq data. FPKM (Fragments Per Kilobase of exon Model) is a measurement of transcript abundance in RNAseq experiments. C) Western blotting and densitometry of β2-chimerin. The bars represent the fold change of the normalized intensity of β2-chimerin (compared to β-actin) versus spleen. The number between parenthesis represent the number of samples.

To identify critical genes targeted by chromosome 7 imbalances (presumably TSG located within CDR and/or oncogene(s) harbored by CGR) in HSTL, we ran 10 different inference analyses (using the MA and RNAseq data) comparing HSTL *vs* PTCL-NOS, NK/TCL, AITCL, T-ALL, nonsorted normal T-cells, sorted activated γδT-cells, nonmalignant spleen and thymus. Then, we focused on the CDR- and CGR-associated genes. Among the roughly 550 genes located within the CDR, only 17 genes were found dysregulated (Table S6). Of note, *PHF14*, our initial candidate TSG, was not dysregulated in any of the comparisons performed. Surprisingly, the comparison *vs* γδT-cells did not identify any downregulated gene in the CDR. Five genes (*TSPAN13, HDAC9, CHN2, EPDR1* and *TARP*), however, were frequently upregulated. Interestingly, *CHN2* was upregulated in all 10 comparisons (Figure 3A and 3B) with a 15 FC in the comparison *vs* γδT-cells (Table S6). The CGR comprises approximately 650 genes. Twenty nine of these genes were dysregulated, including 13 (44.8%) which were exclusively upregulated, four (13.8%) which were downregulated and 12 (41.3%) showing a heterogeneous pattern of expression. The 13 upregulated genes included *ABCB1, RUNDC3B, KRIT1, SAMD9, SGCE, PEG10, PPP1R9A, ZNF655, PILRB, NAPEPLD, PUS7, PIK3CG* and *NRCAM*. Except for *NRCAM*, all these genes were upregulated in HSTL *vs* γδT-cells. Notably, *ABCB1, RUNDC3B* and *PPP1R9A* were upregulated in all 10 comparisons.

To unravel gene expression profile and gene signature of HSTL, we searched for dysregulated genes genomewide and focused on 401 genes which were up- or downregulated in at least four comparisons (Table S7). This 401 geneset, as well as genes found to be dysregulated in individual comparisons, were further explored using Ingenuity Pathway Analysis (IPA). As shown in Table 2, the 401 genes dysregulated in HSTL are implicated in important biological processes, pathways and diseases, including cancer (262 molecules) and inflammatory diseases (94 molecules). “Natural Killer Cell signaling” surfaced as the top canonical pathway dysregulated in HSTL. This pathway was also significantly dysregulated when HSTL was compared with activated γδT-cells, NK/TCL, AITCL, PTCL-NOS and spleen. Notably, the canonical pathway “Role of NFAT in regulation of the immune response” was the second and third top dysregulated pathway in HSTL *vs* activated γδT-cells (Figure S3) and *vs* nonmalignant spleen, respectively. Details of individual IPAs can be found in Figure S4.

**Table 2:** Ingenuity Pathway Analysis: Most significant networks, functions and pathways associated to the top 401 genes differentially expressed in HSTL.

**Top Networks**		
Associated Network Functions	**Score**	
Cellular Assembly and Organization, Hematological System Development and Function, Inflammatory Response	40	
Digestive System Development and Function, Embryonic Development, Organismal Development	40	
Cell Morphology, Cellular Assembly and Organization, Carbohydrate Metabolism	38	
Connective Tissue Disorders, Developmental Disorder, Skeletal and Muscular Disorders	37	
Cancer, Cell Morphology, Organ Morphology	33	
**Top Diseases and Bio Functions**		
**Name**	**p-value**	**No of molecules**
Cancer	3.08E-20 -3.87E-05	265
Reproductive System Disease	2.90E-16 -3.95E-05	104
Connective Tissue Disorders	3.43E-15 -1.54E-05	73
Inflammatory Disease	3.43E-15 -2.37E-05	95
Skeletal and Muscular Disorders	3.43E-15 -3.35E-05	83
**Molecular and Cellular Functions**		
**Name**	**p-value**	**No of molecules**
Cellular Development	1.98E-23 -2.92E-05	186
Cellular Growth and Proliferation	1.98E-23 -3.32E-05	180
Cellular Movement	6.53E-21 -3.62E-05	126
Cell Morphology	5.60E-19 -2.92E-05	129
Cell-To-Cell Signaling and Interaction	1.12E-15 -2.69E-05	148
**Physiological System Development and Function**		
**Name**	**p-value**	**No of molecules**
Tissue Morphology	2.19E-20 -2.00E-05	141
Hematological System Development and Function	5.86E-20 -3.62E-05	144
Immune Cell Trafficking	7.14E-16 -3.62E-05	95
Embryonic Development	6.66E-14 -2.35E-05	96
Lymphoid Tissue Structure and Development	6.66E-14 -2.84E-05	73
**Top Canonical Pathways**		
**Name**	**p-value**	**Ratio**
Natural Killer Cell Signaling	4.36E-07	13/118 (0.11)
Crosstalk between Dendritic Cells and Natural Killer Cells	1.73E-06	11/106 (0.104)
Granulocyte Adhesion and Diapedesis	4.54E-05	13/181 (0.072)
Agranulocyte Adhesion and Diapedesis	8.03E-05	13/191 (0.068)
Hepatic Fibrosis/Hepatic Stellate Cell Activation	4.38E-04	10/155 (0.065)

Considering the important role of Nuclear factor of activated T cells (NFAT) transcription factors in T-cell biology and cancer [26]–[29], we additionally analyzed expression pattern of genes encoding proteins belonging to the large lincRNA-protein complex [also known as the Non-coding RNA Repressor Of NFAT (NRON) complex], recently found to be associated with NFAT [30], [31], and the kinase LRRK2 which is linked to this complex as a negative regulator of NFATC2 [32], [33]. Several of these genes were found dysregulated in HSTL *vs* γδT-cells (Table S7). Particularly interesting was the finding of *LRRK2* upregulation in 4 of 10 comparisons performed (FC = 31.25 in HSTL *vs* normal T-cells, FC = 22.77 in HSTL *vs* δγT-cells, FC = 9.22 in HSTL *vs* non-malignant thymus, and FC = 9.03, in HSTL *vs* T-ALL). In contrast, *CAMK4* was significantly downregulated in HSTL in 7 of the 10 inference analyses performed (Table S7, Figure S5). Calcium/calmodulin dependent kinase 4 (encoded by *CAMK4*) binds Ca2+/calmodulin in the cytoplasm [34]. In the nucleus, CAMK4 regulates, mainly by phosphorylation, the activity of several transcriptional activators, including NFATC2 [34], [35]. Other interesting gene emerging in our study is *ITGAD*, found to be upregulated in HSTL. It encodes the integrin AlphaD, a member of a family of molecules implicated in immunological synapse formation, cell-matrix adhesion, integrin-mediated signaling pathway and proliferation of activated T-cells [36].

In addition, we analyzed expression pattern of chromosome 8-associated genes. Seven genes, *ANGPT1, CA1, CA2, SLC25A37, TOX,* and *MYBL1*, were found upregulated in at least four comparisons. The *MYC* oncogene was upregulated only in the comparison of HSTCL *vs* γδT-cells (FC = 7.01; FDR_BH = 0.0011).

**Figure 4:**
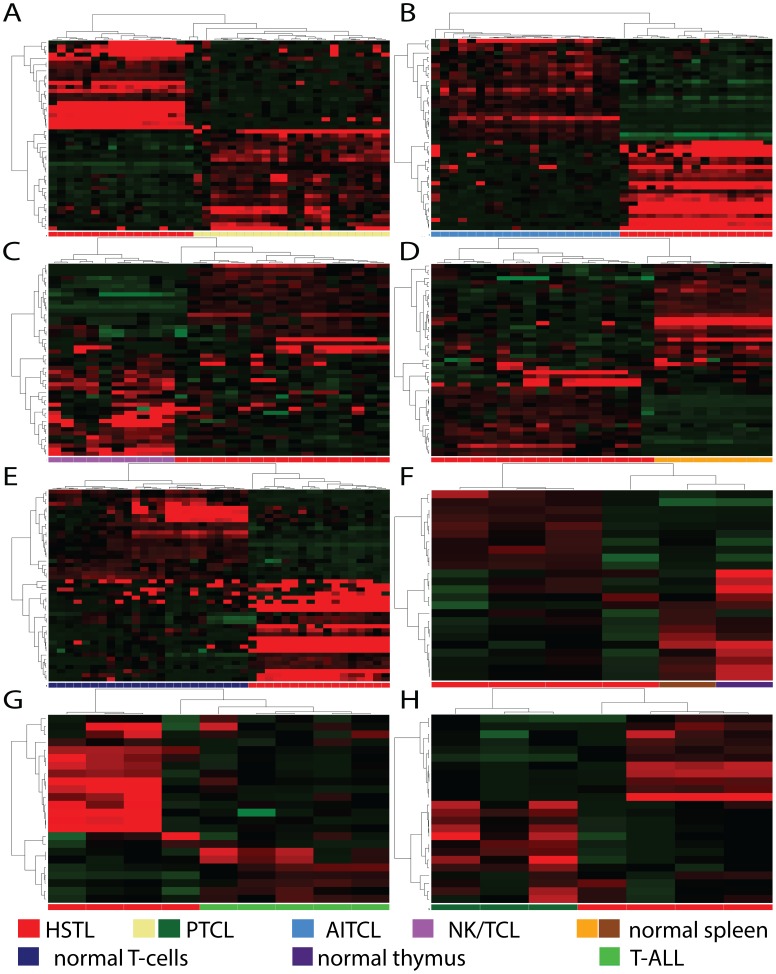
Hierarchical clustering using the 24 gene signature for HSTL. The MA data (A–E) and RNAseq data (F–H) show an accurate separation of the HSTL cluster from PTCL, AITCL, NK/TCL, nonmalignant spleen, normal T-cells, nonmalignant thymus and T-ALL.

To build the gene signature for HSTL several unsupervised hierarchical cluster analyses were performed to find the minimal number of genes that keep the integrity of the HSTL samples cluster in all the comparisons (see Materials and Methods). This yielded a list of 24 genes, including 11 upregulated and 13 downregulated (Table 3). IPA showed that the vast majority of these genes is involved in ‘Cancer’, ‘Cellular growth and proliferation’, ‘Cell death and survival’ and ‘Cell-to cell signaling ’ (Table S8). Using this geneset, HSTL was distinct from AITCL, NK/TCL, PTCL, nonmalignant spleen and T-cells in the MA data, and from PTCL, T-ALL, nonmalignant spleen and thymus in the RNAseq data (Figure 4, Figure S6).

**Table 3:** List of genes comprising the HSTL signature.

Gene Symbol	Chromosome	Position	Name	Location*	Type molecules
**Upregulated**					
*ABCB1*	7	87133175	ATP-binding cassette, sub-family B (MDR/TAP), member 1	PM	transporter
*CD200R1*	3	112640056	CD200 receptor 1	PM	other
*CD5L*	1	157800704	CD5 molecule-like	PM	transmembrane receptor
*ITGAD*	16	31404633	integrin, alpha D	PM	other
*PPP1R9A*	7	94536948	protein phosphatase 1, regulatory subunit 9A	PM	other
*S1PR5*	19	10623623	sphingosine-1-phosphate receptor 5	PM	G-protein coupled receptor
*TMEM178A*	2	39892122	transmembrane protein 178A	PM	other
*CHN2*	7	29234120	chimerin (chimaerin) 2	C	other
*CHSY3*	5	129240165	chondroitin sulfate synthase 3	C	enzyme
*FCRLB*	1	161691353	Fc receptor-like B	C	other
*PRDM16*	1	2985732	PR domain containing 16	N	transcription regulator
**Downregulated**					
*CCR7*	17	38 710021	chemokine (C-C motif) receptor 7	PM	G-protein coupled receptor
*CD200*	3	112051915	CD200 molecule	PM	other
*CD28*	2	204571198	CD28 molecule	PM	transmembrane receptor
*CD5*	11	60869867	CD5 molecule	PM	transmembrane receptor
*CD83*	6	14117872	CD83 molecule	PM	other
*CXCR3*	X	70835766	chemokine (C-X-C motif) receptor 3	PM	G-protein coupled receptor
*GPR183*	13	99946784	G protein-coupled receptor 183	PM	G-protein coupled receptor
*SLAMF1*	1	160577890	signaling lymphocytic activation molecule family member 1	PM	transmembrane receptor
*FAM134B*	5	16473147	family with sequence similarity 134, member B	C	other
*GZMK*	5	54320081	granzyme K (granzyme 3; tryptase II)	C	peptidase
*CAMK4*	5	110559351	calcium/calmodulin-dependent protein kinase IV	Nucleus	kinase
*PRRX1*	1	170631869	paired related homeobox 1	Nucleus	transcription regulator
*CCL19*	9	34689564	chemokine (C-C motif) ligand 19	ES	cytokine

*PM, plasma membrane; C, cytoplasm; N, nucleus; ES, extracellular space.

### Validation studies

**Figure 5:**
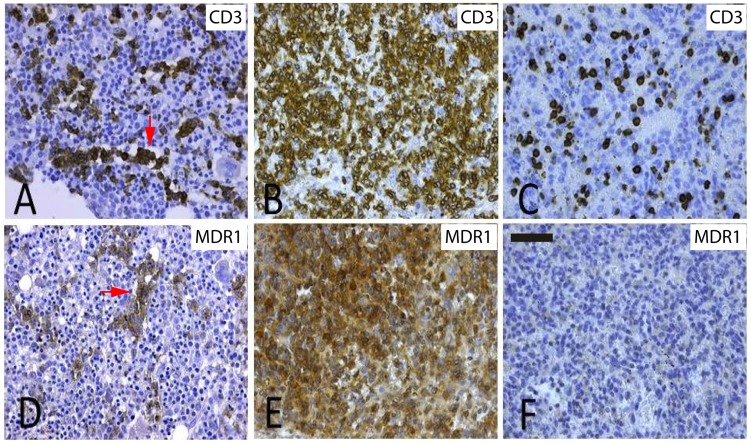
Morphology and ABCB1 expression in HSTL cases detected by IHC. Immunohistochemical stainings (A–C, anti-CD3; D-F, anti-MDR1/ABCB1) of the typical intrasinusoidal spread (red arrows) by HSTL cells in the bone marrow (A/D, case 4) and spleen (B/E, case 6), respectively, compared to staining pattern in normal spleen (C/F). Pictures captured by Leica DFC290HD camera at 400X. Scale bar  = 50 µm.

Expression value of six genes, *CHN2, ITGAD, CAMK4, PEG10, PPP1R9A* and *NFATC2*, were validated by QRT-PCR performed on cases 4, 5, 7 and sorted γδT-cells (Figure S5). The analysis confirmed downregulation of *CAMK4* and *NFATC2* in all cases and showed upregulation of the remaining genes in at least two out of three cases analyzed, when compared with γδT-cells. Western blotting was applied to demonstrate expression of the *CHN2-*encoded β2-chimerin in four index cases (Table 1). As γδT-cells were not available, nonmalignant spleen was used as control. In addition, we analyzed Jurkat T-cells (positive control) [37] and i(7)(q10)-positive T-cell lymphoblastic lymphoma (Figure 3C). Β2-chimerin was detected in all samples analyzed and its expression level in HSTL was higher than in spleen and ≥ than in Jurkat T-cells known to overexpress *CHN2*. The expression of the ABCB1 protein was demonstrated by IHC (Figure 5).

### Mutation and fusion genes analysis

The obtained RNAseq data were subjected to mutation and gene fusion analysis. Three genes were found mutated in all 4 HSTL cases analyzed: *SEPT7* (7p14.2), *MAP4K5* (14q22.1) and *CYTH2* (19q13.33) (Table S9). However, all these mutations were predicted as benign or tolerated by VEP. Deleterious mutations were random (e.g. mutation of *ATM* in case 4). We did not detect any nonrandom indel except for Del-3′UTR (-/TCTC, chr7:29,550,568-29,550,571) in the *CHN2* gene. This deletion, however, was reported as SNP (rs71800296).

DeFuse revealed only four fusions, which were absent in nonmalignant spleen and thymus, and occurred in at least two cases (Table S10). Two of these fusions, LSP1->AC027612.6 and LL22NC03-80A10.6->RP11-236F9.4, involve either a gene with its pseudogene or two pseudogenes, respectively. The third fusion involves a sequence near an uncharacterized noncoding RNA (LOC402483) on chromosome 7q and a sequence (FLJ45340) on the telomere of chromosome 5q without gene annotation by UCSC or RefSeq. The fourth fusion, SEMA4D->RP11-156P1.3, was found in cases 4, 7 and DERL-2. An alternative fusion analysis performed with ArrayStudio did not reproduce the above mentioned fusions but identified four recurrent fusions (≥ two samples). Further analysis using the UCSC Blat tool suggested, however, that these fusions could be false positive predictions since at least one of the involved sequences align to the genome multiple times with 100 percent identity.

## Discussion

Isochromosome 7q is a primary chromosomal aberration in HSTL detected in almost all affected individuals. The contribution of this aberration to the pathogenesis of disease is still unknown. Recent identification of r(7), a rare variant aberration in HSTL [11], [13], provides an unique opportunity to narrow down the critical 7p/7q regions and identify the targeted genes. We set up a collaborative in-depth genomic study of six HSTL cases with i(7)(q10), including the DERL-2 cell line, and three cases with r(7). Using high resolution aCGH, we profiled all samples and defined a CDR (34.88 Mb) at 7p22.1p14.1 (3.48–38.36 Mb) and a CGR (38.77 Mb) at 7q22.11q31.1 (86.12–124.89 Mb) (Figure 1A). Interestingly, CGR encompasses a region of 13 Mb (86.25–99.27 Mb) selectively amplified in all three cases with r(7). In addition, aCGH mapped the r(7)-associated breakpoints within the *TCRG* (7p14.1) and *TCRB* (7q32) gene clusters, what suggests that r(7) is a byproduct of illegitimate somatic rearrangements of both loci. This defect results in an aberrant *TCRG-TCRB* lesion, formation of r(7) and a consequent loss of the distal 7p/7q regions (Figure 1B and Figure 2). Of note, similar inter-*TCR* rearrangements feature patients with chromosome instability syndromes [38]. We presume that formation of r(7) in γδT-cells was a primary event which was latter followed by a gradual gain of 7q sequences (Figure 2).

The compilation of all gained genomic data led us to hypothesize that loss of 7p22.1p14.1 is the critical pathogenetic event contributing to development of HSTL, while gain of 7q22.11q31.1 provides growth advantages and contributes to chemoresistance of the tumor. Significance of the former imbalance is supported by the cytogenetic finding of der(7)t(7;15)(p22;q21) associated with loss of 7p22pter (breakpoint not validated by FISH/aCGH) but not affecting 7q, in one case of i(7)(q10)-negative HSTL [14]. Chromosomal deletions, especially homozygous deletions, are considered as hallmarks of TSG localization in cancer cells [39]. Therefore, identification of a biallelic 7p21.3 microdeletion encompassing *PHF14*, a postulated TSG, in case 4 seemed to be the groundbreaking finding of the study. Particularly, that *PHF6*, other member of the PHF gene family, plays a role of tumor suppressor in T-ALL [40]. Subsequent investigations, including sequencing and MA/RNAseq analysis, however, did not provide any evidence of inactivated mutation(s) or downregulated expression of *PHF14* in other HSTL cases.

To further attempt identification of genes targeted by i(7)(q10)/r(7), we performed an integrative genomic and transcriptomic analysis initially focusing on genes located within the common deleted (3.48–38.36 Mb) and gained (86.12–110.19 Mb) regions. Surprisingly, none of the CDR-associated genes was significantly and recurrently downregulated, however, one gene, *CHN2*, appeared to be commonly upregulated in HSTL. *CHN2* encodes β2-chimerin which displays GTPase-activating protein activity and is involved in small GTPase mediated signal transduction [41], [42]. Β2-chimerin is ubiquitously expressed in T-lymphocytes and engaged in the regulation of chemokine-modulated responses [41]. Recent studies implicate β2-chimerin in the downmodulation of RAC1 (ras-related C3 botulinum toxin substrate 1) activity during T-cell synapse formation and suggest its contribution to diacylglycerol-mediated regulation of cytoskeletal remodeling during T-cell activation [37], [41], [42]. Given the important role of β2-chimerin in T-cell biology, *CHN2* emerged as a candidate 7p gene targeted by i(7)(q10)/r(7) in HSTL. The molecular mechanism(s) underlying an enhanced expression of the nondeleted *CHN2* locus in HSTL is unclear, but either loss of 7p-associated negative regulators or gain/activation of 7q-associated positive regulators or deregulation of epigenetic effectors may contribute to this process. Interestingly, regulation data from the Encyclopedia of DNA Elements (ENCODE) (https://genome.ucsc.edu/ENCODE/) revealed several transcription factors regulating expression of *CHN2*, including EZH2, which targets the promotor region of *CHN2* (http://genome-euro.ucsc.edu/cgi-bin/hgTracks?hgS_doOtherUser=submit&hgS_otherUserName=JulioFinalet&hgS_otherUserSessionName=regulation%20at%20CHN2%20promotor_simple). EZH2, a catalytic subunit of the Polycomb Repressor Complex 2 [43], is involved in epigenetic transcriptional repression of genes through histone methylation and consequent chromatin condensation. As expression of *EZH2* is significantly downregulated in HSTL *vs* δγT-cells (FC = -3.012; FDR_BH = 0.0284), it may affect expression of *CHN2*. The *EZH2* gene (7q36.1) is not mutated in HSTL, but monoallelically deleted in all r(7)-positive cases.

Integrative analysis of chromosome 7q identified a set of 13 constantly upregulated genes, including *ABCB1, RUNDC3B* and *PPPAR9A*, found to be selectively amplified in cases with r(7). The top candidate is *ABCB1* (alias *MDR1*), already known to be overexpressed in HSTL [5], [44]. *ABCB1* codes a multidrug transporter P-glycoprotein which belongs to the superfamily of ATP-binding cassette (ABC) transporters [45]. These molecules, which function in normal biology to protect cells from harmful toxins and xenobiotics, contribute to drug resistance of cancers by extruding a variety of chemotherapeutic agents from the tumor cells [46]. Amplification, rearrangement and/or overexpression of ABCB1 have been associated with chemotherapy failure in many cancers [47]–[51]. *RUNDC3B* is likely involved in multiple Ras-like GTPase signaling pathways [52], [53] and is implicated in transformation and progression of breast cancer [54]. *PPP1R9A* encodes neurabin 1 which constitutes a regulatory subunit of protein phosphatase I [55]. Neurabin 1 is a multi-functional F-actin-binding protein, and like other phosphatases, is potentially implicated in tumorigenesis [56]. Although upregulation of *PEG10* was not constantly observed in HSTCL, it is worth note that this transcription factor is implicated in tumorigenesis [57]–[59]. *PEG10* is a postulated target of 7q21 amplification in hepatocellular carcinoma [59] and its overexpression in cancer correlates with disease progression, invasiveness and aggressiveness [57], [60]. Altogether, these data support our hypothesis that the i(7)(q10)/r(7)-related duplication or amplification of 7q mainly activates genes which provide growth advantage of lymphoma cells and are responsible for an intrinsic drug resistance and aggressiveness of HSTL.

Gene expression profile of HSTL has been previously investigated by Miyazaki *et al*. (2009) [44] and Travert *et al*. [5]. The first group showed that the TCR-associated gene signature accurately classifies γδHSTL and distinguishes it from PTCL. The latter group demonstrated that HSTL is characterized by a distinct molecular signature, irrespective of the TCR-cell lineage. GEP revealed overexpression of multiple NK-cell-associated molecules and several cancer genes, including *FOS, VAV3*, *S1PR5* and *SYK*. Among the most downregulated genes was a tumor suppressor gene *AIM1*, found to be methylated in HSTL. Results of our transcriptomic analysis performed on altogether 17 HSTL cases (four index cases and 13 previously published cases [5], [61]) are in line with the previous findings. Except for a few transcripts, we found the same differential expression of the vast majority of genes described by Travert *et al*. [5]. In addition, we significantly diminished a number of biomarkers discriminating HSTL from other malignancies to 24. Remarkably, the geneset comprises three chromosome 7 genes located either in CDR or CGR: *CHN2*, *ABCB1* and *PPP1R9A*.

**Figure 6:**
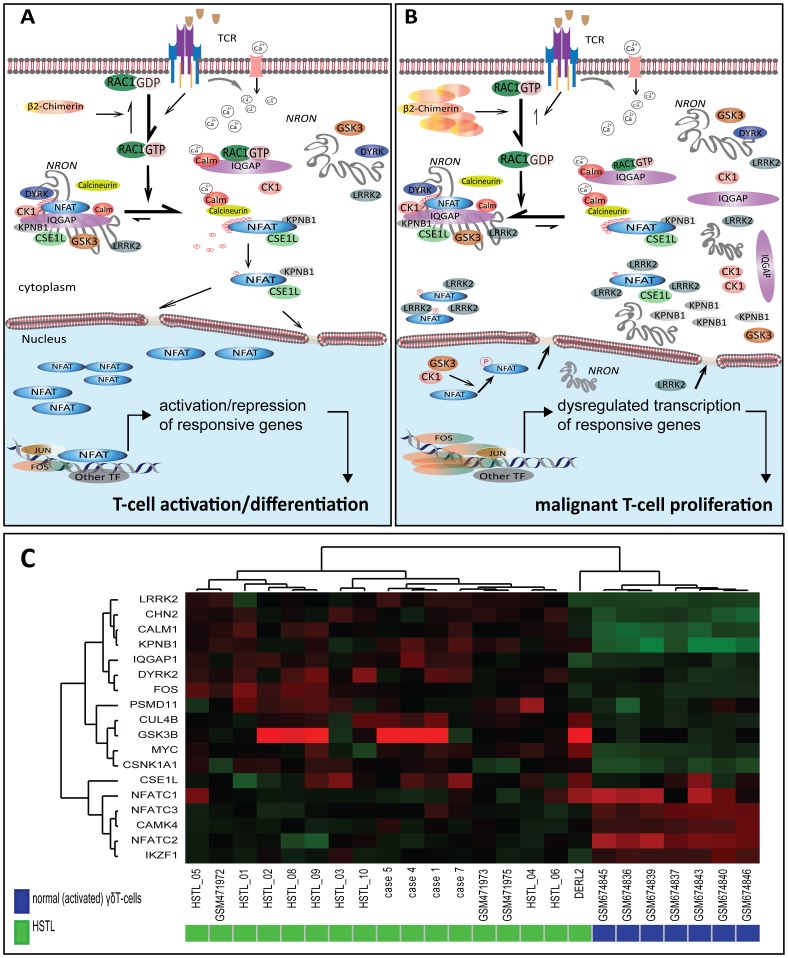
Postulated model for the pathogenesis of HSTL. (A) In resting T-cells, NFAT proteins are located in the cytoplasm and are associated with a large RNA-protein scaffold complex composed of the lincRNA NRON, a repressor of NFAT [30], and several additional proteins [31]. NFAT proteins are heavily phosphorylated through synergistic action of three different family of kinases, casein kinase 1 (CK1), glycogen synthase kinase 3 (GSK3), and dual specificity tyrosine phosphorylation regulated kinase (DYRK) [29]. When T-cells are stimulated, TCR engagement triggers a rapid increase in intracellular calcium (Ca^2+^) and activation of RAC1, a GTPase which belongs to the RAS superfamily of small GTP-binding proteins [79]. The active, GTP-bound RAC1 binds to IQGAP (IQ-domain GTPase-activating protein) negatively regulating its binding affinity for other proteins and consequently, stimulating the disassembly of the NRON complex [31], [82], [83]. In parallel, the calcium increase leads to activation of calmodulin, a calcium-binding messenger protein, which activates of the phosphatase calcineurin. This enzyme dephosphorylates NFAT and promotes nuclear transport of activated NFAT by importins (KPNB1, CSE1L). In the nucleus, NFAT, in synergy with a numbers of other transcriptional regulators (e.g. FOS and JUN), participates in a transcriptional regulation of a wide range of genes involved in immune system responses and organs development [29], [66]. (B) We postulate that formation of i(7)(q10) or r(7) in γδT-cell triggers an aberrant expression of β2-chimerin which subsequently inactivates RAC1 by keeping it in a GDP-bound state. This prevents RAC1 binding with IQGAP resulting in a strengthening of the NRON complex and arrests the phosphorylated NFAT in the cytoplasm. Cytoplasmic retention of NFAT may be also attributed to the kinase LRRK2 (overexpressed in HSTL), which blocks the transport of NFAT to the nucleus [32], [33]. The significantly reduced nuclear level of NFAT leads to dysregulated transcription of responsive genes controlling cell-cycle, cell death and proliferation, and eventually, to malignant transformation and clonal proliferation of i(7)(q10)/r(7)-positive γδT-cells. The candidate causative genes include the *MYC* oncogene (↑ in HSTL), known to be repressed by NFAT [62] and the *IKZF1* tumor suppressor gene (↓ in HSTL) which is activated by NFAT [66]. (C) Hierarchical clustering using NFAT-related genes, including components of the NRON complex. Note that all HSTL samples, except for DERL-2, form a distinct cluster apart from the activated γδT-cells.

Interestingly, IPA showed that the canonical pathway “Role of NFAT in regulation of the immune response” is one of the top dysregulated pathways in HSTL. NFAT is a family of transcription factors playing a crucial role in the development and function the immune system [29]. There are five NFAT family members and three of them, NFATC1, -C2 and -C3 are expressed by T-cells and activated in response to TCR engagement. In resting T-cells, NFAT is located in the cytoplasm, in an inactive hyperphosphorylated form, associated with the NRON complex [31] Upon TCR engagement, NFAT disassociates from the complex and is rapidly dephosphorylated by the phosphatase calcineurin (Figure 6A). Activated NFAT translocates to the nucleus, where in cooperation with other transcriptional partners (e.g. FOS and JUN), it regulates transcription of a wide range of genes. NFAT responsive targets include numerous cytokine genes (*IL2, IL3, IL4, IL5* and *IFNG*) and other genes involved in the control of the cell cycle and apoptosis (e.g. *MYC, IKZF1*, *CDKN1A*, *CD40LG*, *FASLG*, *CDK4* and *NR4A1*) [26], [62]–[66]. Recent studies strongly suggest an important role for the Ca^2+^-calmodulin/calcineurin/NFAT signaling in tumor development and progression [27], [28], [63], and postulate that NFAT transcription factors may act either as oncogenes or TSG [67]. The latter function is assigned to *NFATC2 (alias NFAT1* and *NFATp*), a postulated inhibitor of cell proliferation [63], which is significantly downregulated in HSTL (Table S7, Figure S5). Notably, mice deficient in *NFATC2* showed hyperproliferation of lymphocytes, accompanied by a reduction in cell death and an increased cell cycle rate [64], [68]–[71], whereas *NFATC2-/NFATC3*- double knock-out mice developed lymphoproliferative disorder with marked lymphadenopathy and splenomegaly, decreased activation-induced death and impaired Fas ligand induction [72]. Further studies demonstrated that NFATC2 suppresses neoplastic changes in chondrogenesis [73] and displays pro-apoptotic activity in Burkitt lymphoma [74]. Its ectopic expression in NIH3T3 cells results in cell cycle arrest, apoptosis and inhibition of Rasv12-mediated malignant transformation [67]. NFATC2 also controls the cell-cycle progression by repressing expression of the G0–G1 checkpoint kinase CDK4 and cyclins A2, BA, E and F [75]–[77], and induces apoptosis in NIH3T3 fibroblasts in cooperation with the Ras/Raf/MEK/ERK pathway [63]. Recently, it was reported that haploinsufficency of NFATC2 contributes to the pathogenesis of essential thrombocythemia with del(20q) [78]. In this context, particularly interesting is the study of Caloca *et al*. (2008) linking the Ca^2+^-calmodulin/calcineurin/NFAT pathway with β2-chimerin [37]. The authors showed that an experimental overexpression of β2-chimerin in Jurkat T-cells stimulated by anti-CD3 antibodies significantly inhibits the transcriptional activity of NFATC2. This is caused by β2-chimerin-mediated reduction in the levels of active, GTP-bound, RAC1. As demonstrated previously, activated RAC1 modulates calcineurin and consequently, regulates nuclear import and transcriptional function of NFAT [79].

Based on the published data summarized above, IPA (gene interactions and pathways) and our own data, we propose a hypothetical model for the molecular pathogenesis of HSTL (Figure 6A-B). We presume that defects in the NFAT pathway, reflected by the i(7)(q10)/r(7)-associated overexpression of *CHN2*/β2-chimerin, downregulation of NFATC2 and dysregulation of several NFAT/NRON-related genes (Figure 6C), may collectively lead to a downmodulation of the transcriptional activity of NFATC2. This ultimately results in a transcriptional dysregulation of NFATC2 targets, including genes controlling cell cycle, cell death and proliferation [e.g., *MYC* (↑) and *IKZF1* (↓)], and likely *NFATC2* itself, and eventually to malignant proliferation of γδT-cells.

In summary, our study provides further insight on the genetics and the pathogenic mechanisms of HSTL. We proved that HSTL cases harboring either a typical i(7)(q10) or variant r(7) are characterized by a constant loss of 7p22.1p14.1 and gain of 7q22.11q31.1. As RNAseq has not identified any disease-defining mutations and/or gene fusions, chromosome 7 imbalances remain the only driver genetic events found in this tumor. Based on the integrated genomic and transcriptomic data, we hypothesize that loss of 7p sequences is critical for the development of HSTL. This aberration associates with an enhanced transcription of *CHN2* and overexpression of β2-chimerin, what likely affects the NFATC2 related pathway and leads to a proliferative response. On the other hand, gain of 7q correlates with upregulation of several genes, including *ABCB1*, *RUNDC3B* and *PPP1R9A*, providing growth advantage to malignant cells and contributing to their intrinsic chemoresistance and aggressiveness. The latter process is probably also enhanced by genes activated by the frequently acquired trisomy 8, the set of dysregulated molecules previously discussed by Travert et al. (2012) [5] and by an impaired immune synapse formation in neoplastic δγT-cells caused by an overexpressed β2-chimerin and a downmodulated RAC1 [80]. The proposed here model of the pathogenesis of HSTL needs experimental validation. Further studies are also required to determine whether the mechanism(s) underlying the i(7q)/r(7)-driven pathogenesis of human HSTL are related to the id3-driven neoplastic transformation of murine γδT-cells [81].

## Supporting Information

Figure S1 **Unsupervised Spectral Map Analysis using the microarray data.** Samples are separated according to their original classification (A) regardless of the lab of origin (B). The dots in grey (“variable”) represent microarray probes. Note that HSTL separates from other T/NK cell malignancies and cluster near the spleen samples (reflecting the tissue of origin). The spreading across component one of some HSTL samples is related to the purity of the samples (C). Note that HSTL cases with sorted lymphoma cells cluster near the sorted normal T-cells. Interpretation of this analysis is similar to a principal component analysis (details in: www.vetstat.ugent.be/workshop/Nairobi2004/Bijnens/Bijnens2004.pdf). The values between parentheses in the axes mean the percentage of the total number of variables (here, microarrays probes) that contributes to the variance in a given direction (or component).
(TIF)

Figure S2 **Principal Component Analysis (PCA) using the RNAseq data.** The HSTL samples cluster separately from the T-ALL, PTCL and spleen samples. The values between parentheses in the axes mean the percentage of the total number of variables (here, microarrays probes) that contributes to the variance in a given direction (or component).
(TIF)

Figure S3 **IPA canonical pathway “Role of NFAT in regulating the immune response”: HSTL** ***vs*** **δγT-cells.** The fold change values from the inference analysis of HSTL *vs* δγT-cells were overlaid in this pathway. The red color reflects a positive fold change (in this case, upregulation in HSTL as compared to δγT-cells) and green means negative fold change. Double circles represent a complex of molecules and a green to red gradient means that some components in the complex are downregulated while others are upregulated.
(TIF)

Figure S4 **Top dysregulated canonical pathways resulting from individual analysis in IPA.** The bold numbers mean the number of molecules involved in a given pathway. The percentage value on the top of the graph means the percentage of dysregulated molecules from the total number of molecules involved in the pathway. Pathways for a given analysis are ranked from higher to lower statistical significance. The statistical significance (p-value) of a given pathway is calculated considering the percentage of dysregulated molecules in the pathways, as well as the fold change of dysregulation.
(PDF)

Figure S5 **Expression of selected genes analyzed by QRT-PCR.** The Y-axis represents the fold change of normalized mRNA expression compared to δγT-cells.
(TIF)

Figure S6 **High resolution images of hierarchical clustering using the 24 gene signature for HSTL.** The dendograms were generated using the Pearson correlation to calculate the distance and a complete link. The associated heatmap was normalized using a robust center scale.
(PDF)

Table S1 **List of FISH probes.**
(XLSX)

Table S2 **List of primers used for sequencing and QRT-PCR.**
(XLSX)

Table S3 **List of cases included in the expression microarray analysis.**
(XLSX)

Table S4 **Segment report from the aCGH data.**
(XLSX)

Table S5 **Aligment report of RNAseq analysis of HSTL, PTCL, spleen and thymus.**
(XLSX)

Table S6 **Dysregulated genes in CDR (7p) and CGR (7q).**
(XLSX)

Table S7 **Genomewide dysregulated genes in 10 comparisons (XLSX).**
(XLSX)

Table S8 **IPA functional annotation of genes included in the HSTL signature.**
(XLSX)

Table S9 **Annotated mutations found in the index cases analyzed by RNAseq.**
(XLSX)

Table S10 **Results of the gene fusion analysis.**
(XLSX)
