# Supplementary figures and images for: Integrative Genomic and Transcriptomic Analysis Identified Candidate Genes Implicated in the Pathogenesis of Hepatosplenic T-Cell Lymphoma
Source: PLoS One. 2014 Jul 24;9(7):e102977. doi: 10.1371/journal.pone.0102977 (PMC4109958; doi:10.1371/journal.pone.0102977)

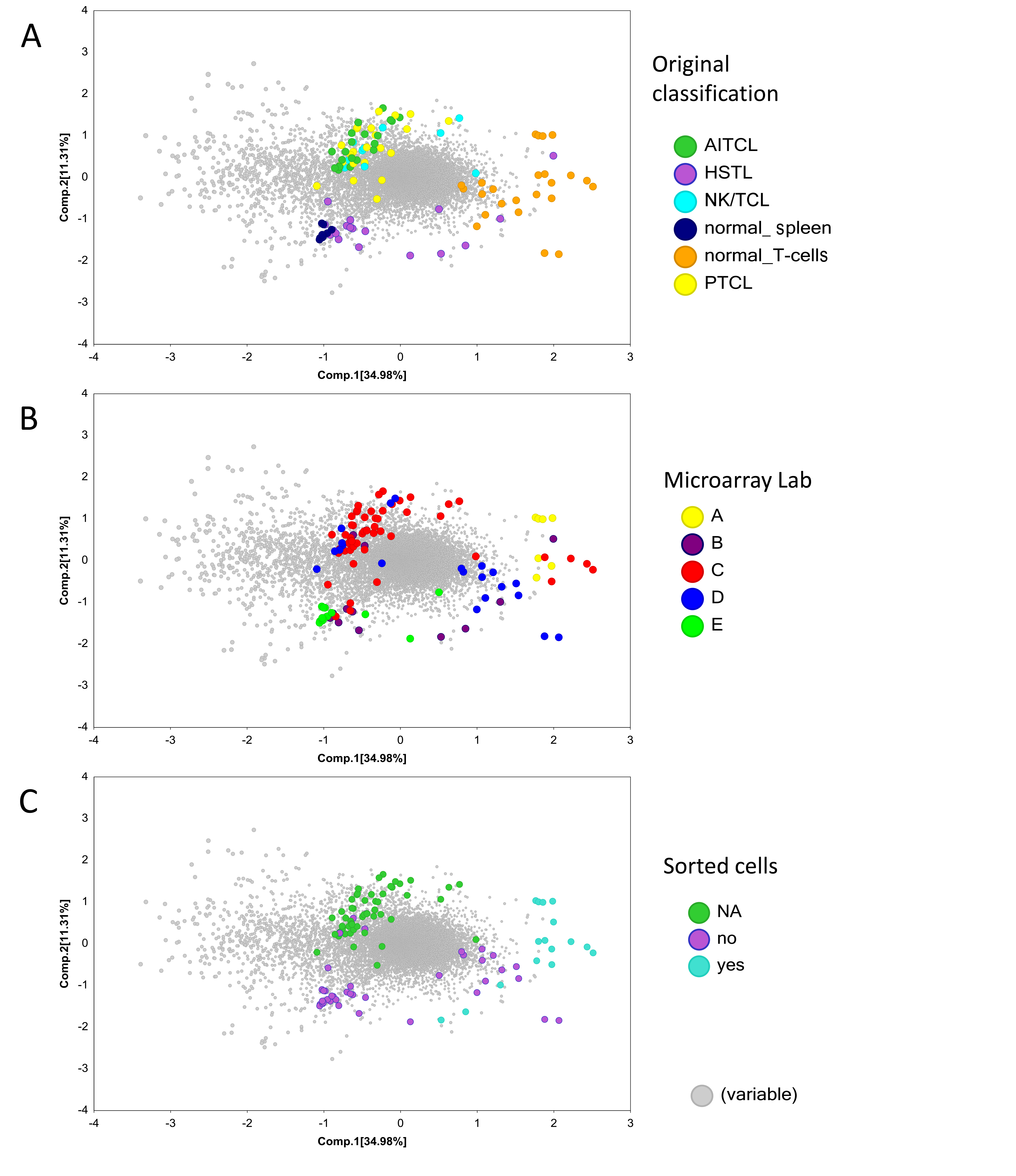

Supplement: Figure S1 — Unsupervised Spectral Map Analysis using the microarray data. Samples are separated according to their original classification (A) regardless of the lab of origin (B). The dots in grey (“variable”) represent microarray probes. Note that HSTL separates from other T/NK cell malignancies and cluster near the spleen samples (reflecting the tissue of origin). The spreading across component one of some HSTL samples is related to the purity of the samples (C). Note that HSTL cases with sorted lymphoma cells cluster near the sorted normal T-cells. Interpretation of this analysis is similar to a principal component analysis (details in: www.vetstat.ugent.be/workshop/Nairobi2004/Bijnens/Bijnens2004.pdf). The values between parentheses in the axes mean the percentage of the total number of variables (here, microarrays probes) that contributes to the variance in a given direction (or component). (TIF) [file pone.0102977.s001.tif]

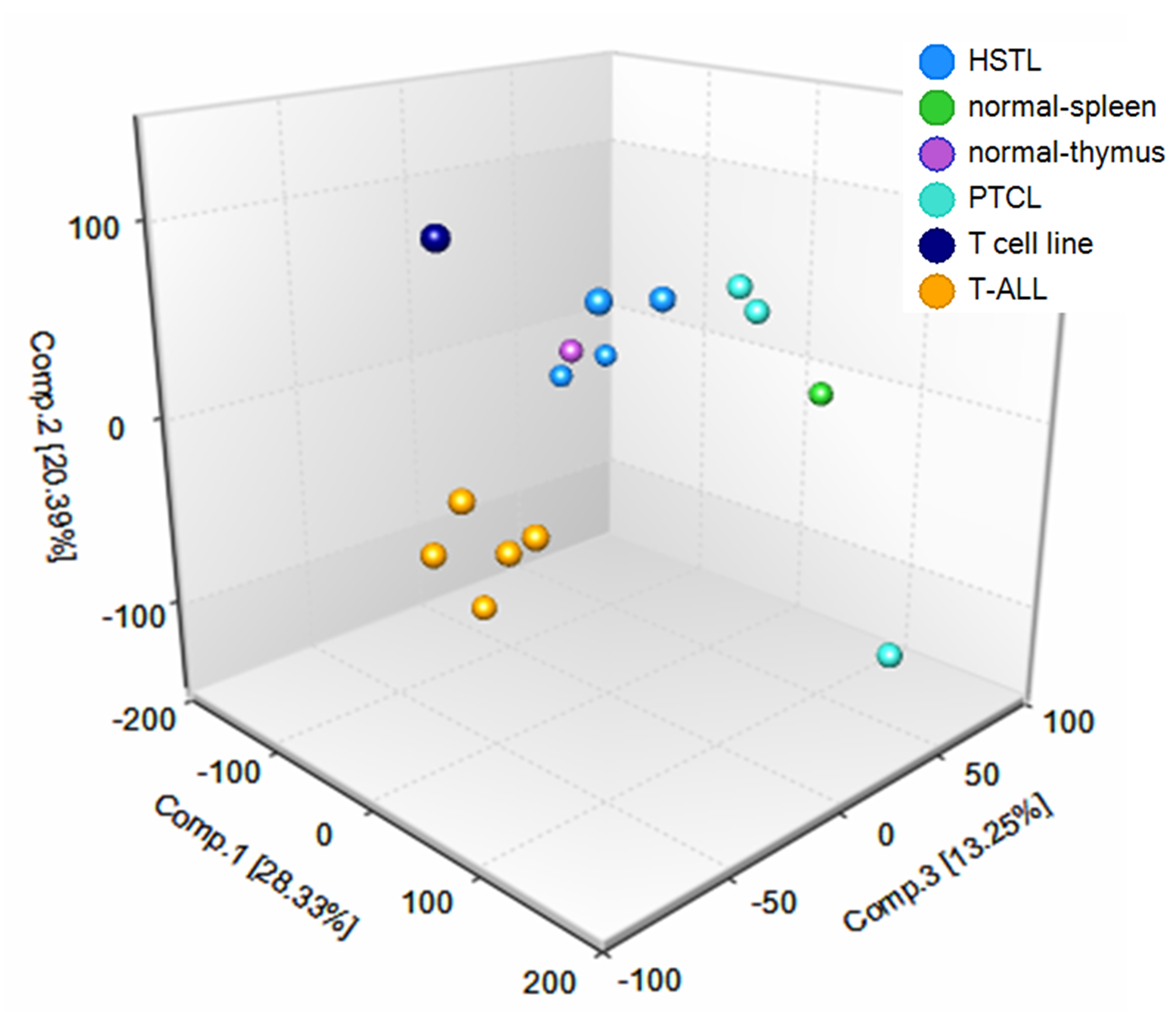

Supplement: Figure S2 — Principal Component Analysis (PCA) using the RNAseq data. The HSTL samples cluster separately from the T-ALL, PTCL and spleen samples. The values between parentheses in the axes mean the percentage of the total number of variables (here, microarrays probes) that contributes to the variance in a given direction (or component). (TIF) [file pone.0102977.s002.tif]

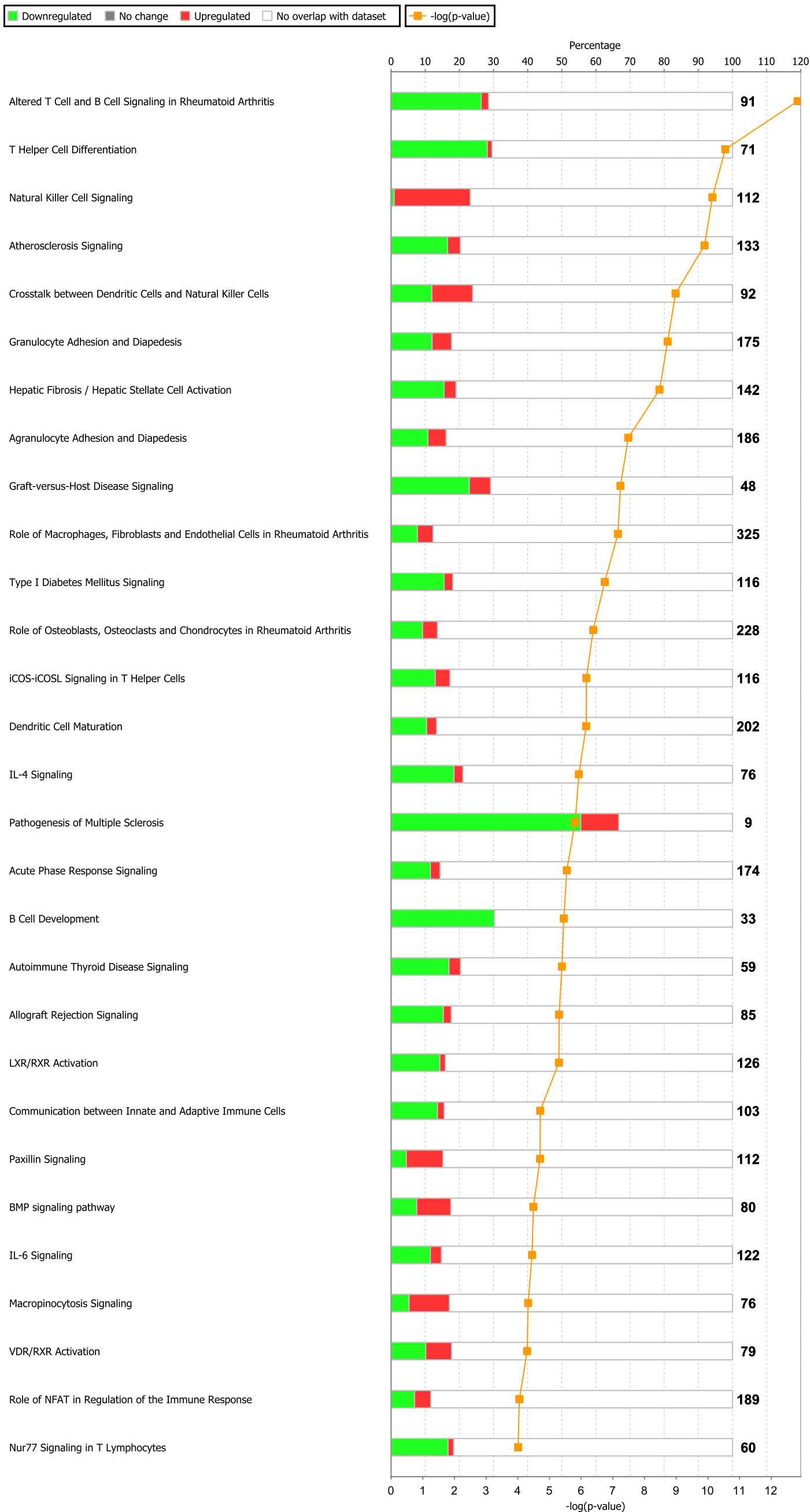

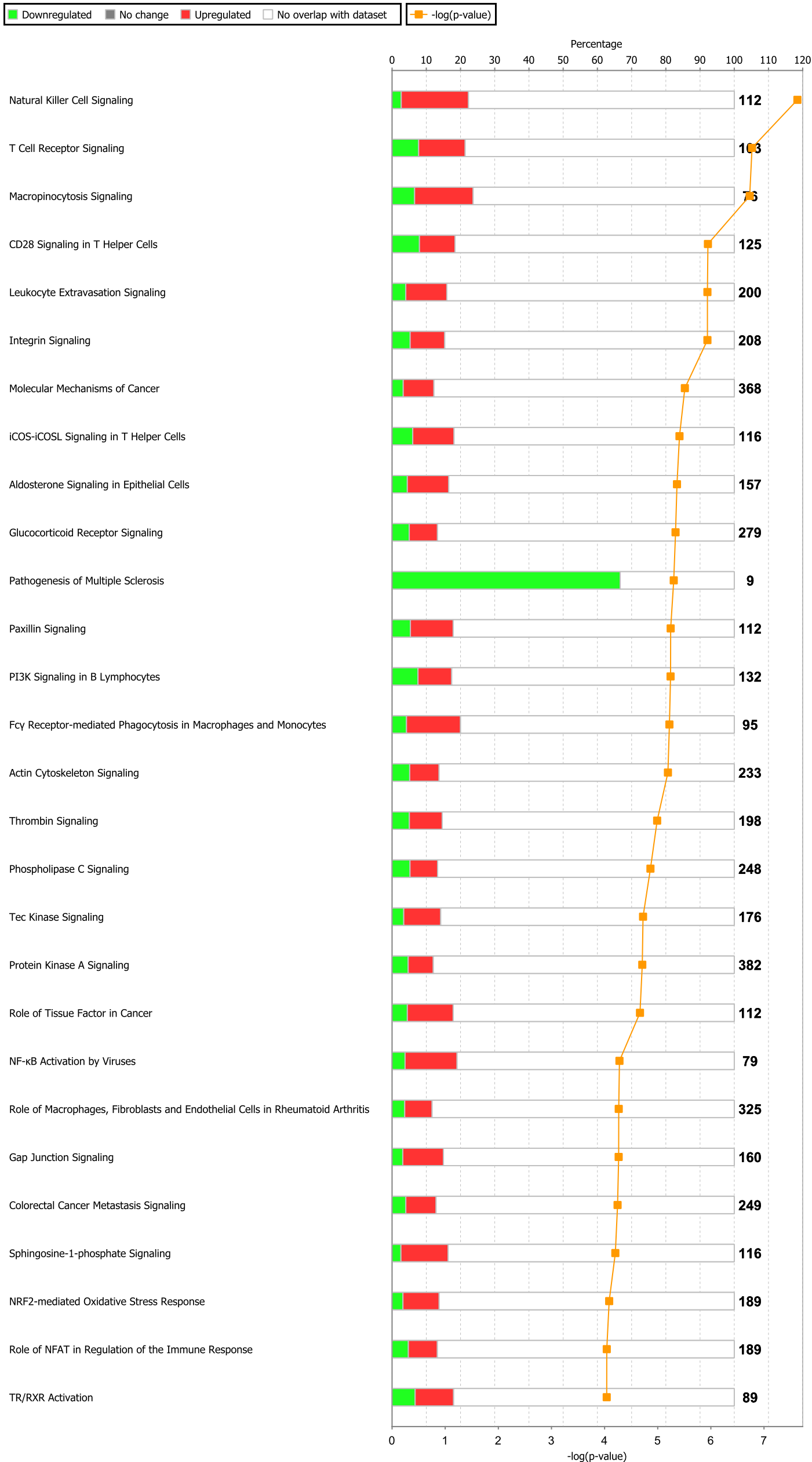

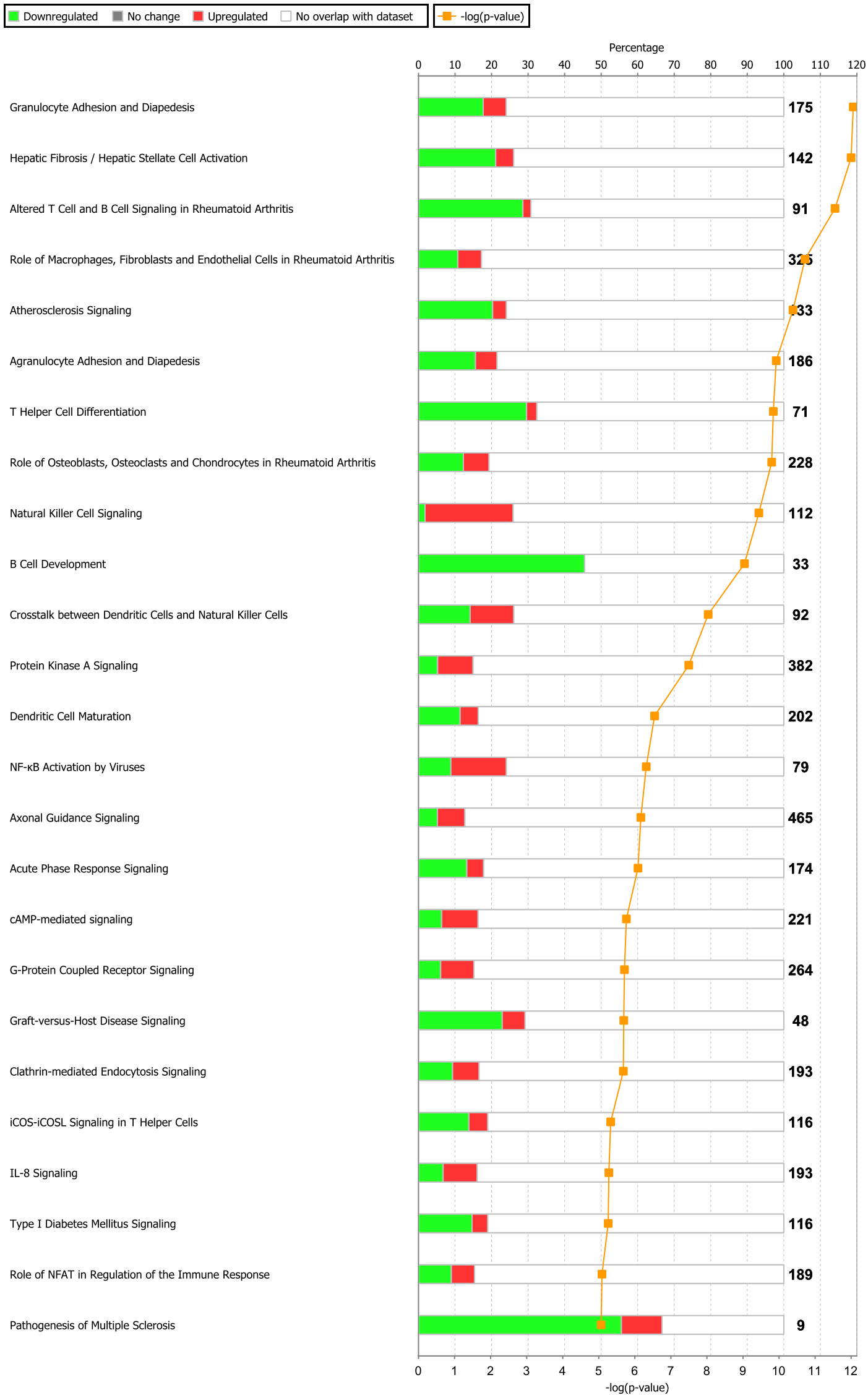

© 2000-2014 Ingenuity Systems, Inc. All rights reserved.

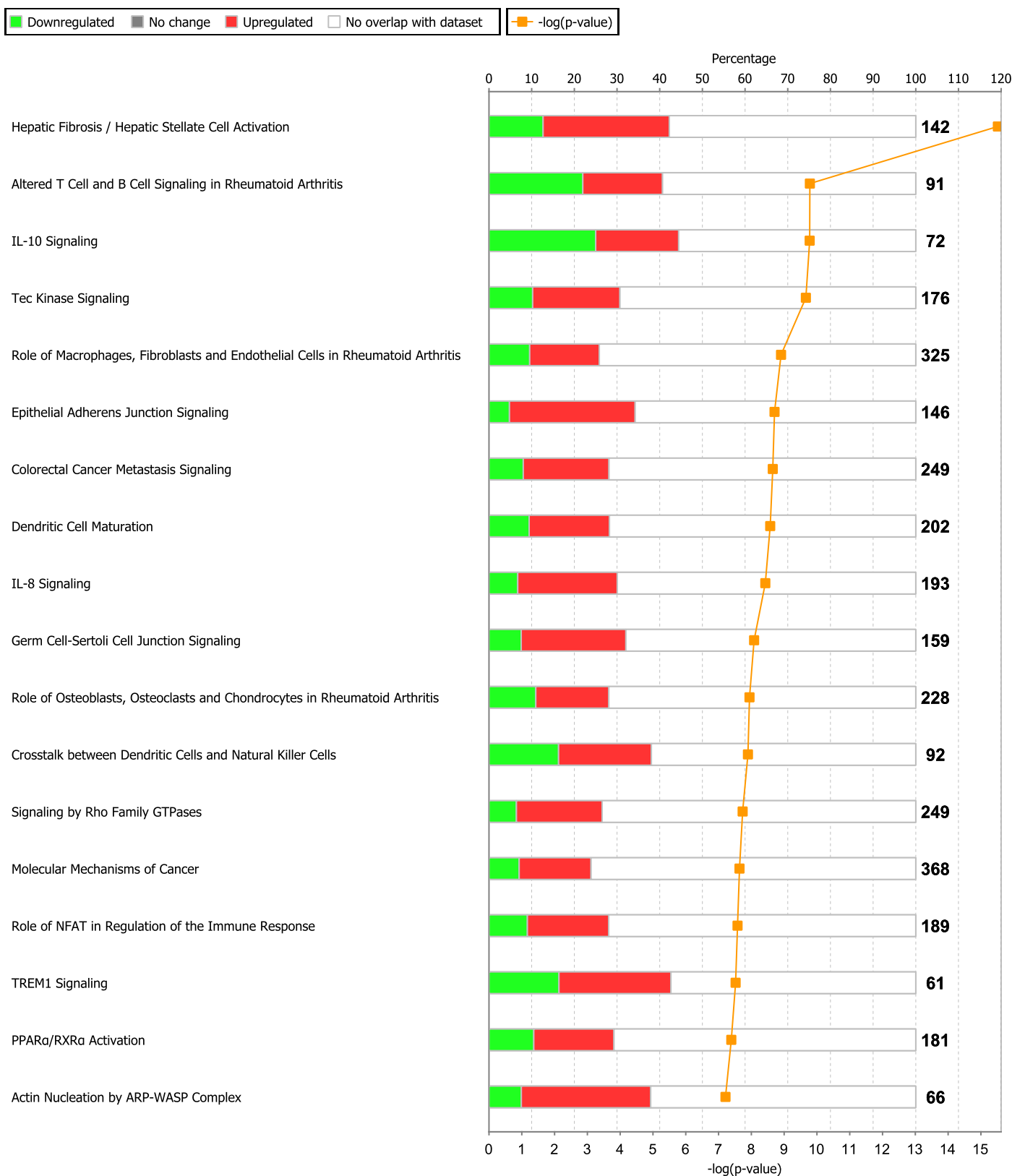

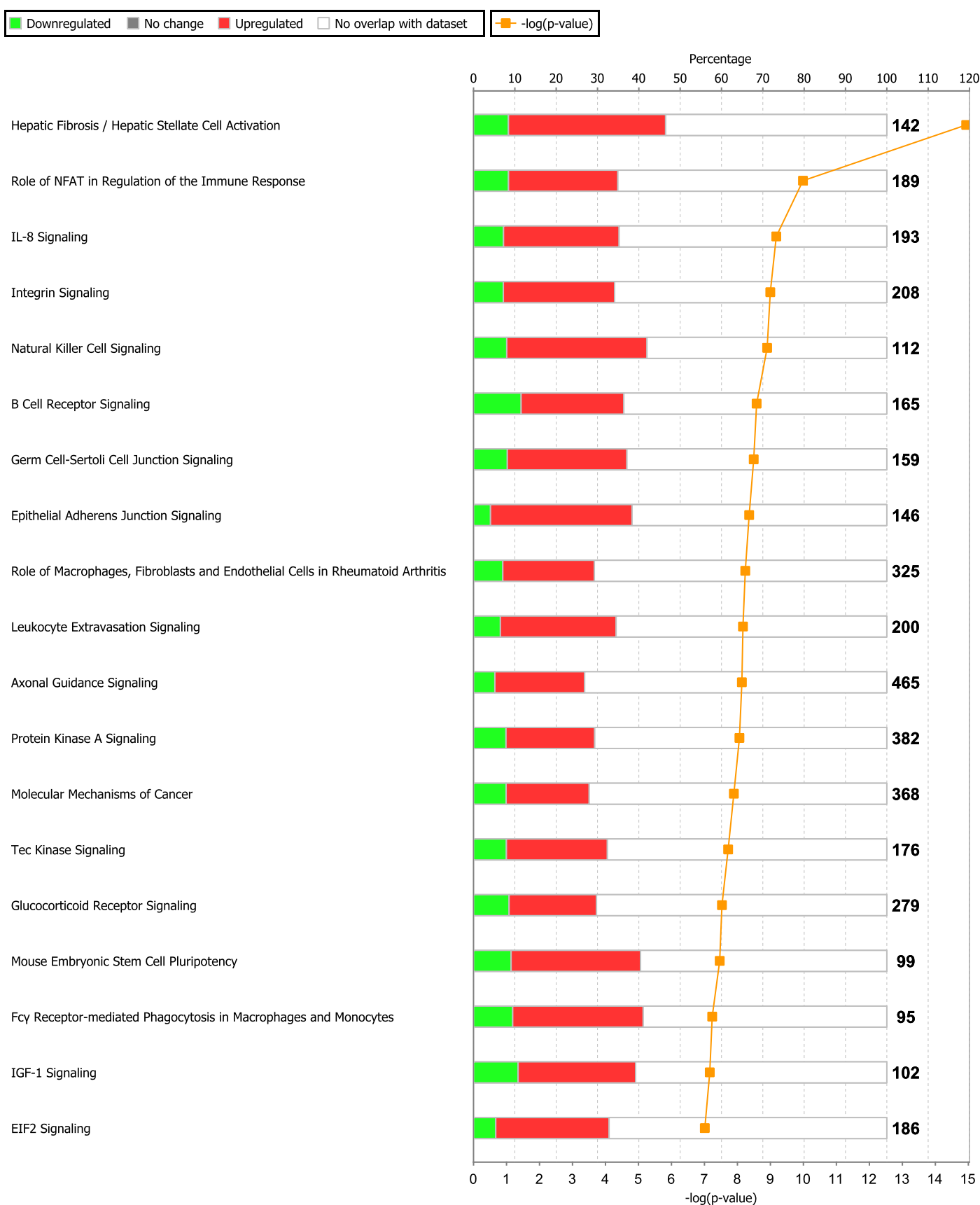

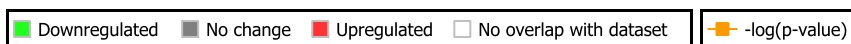

## HSTL vs. normal spleen

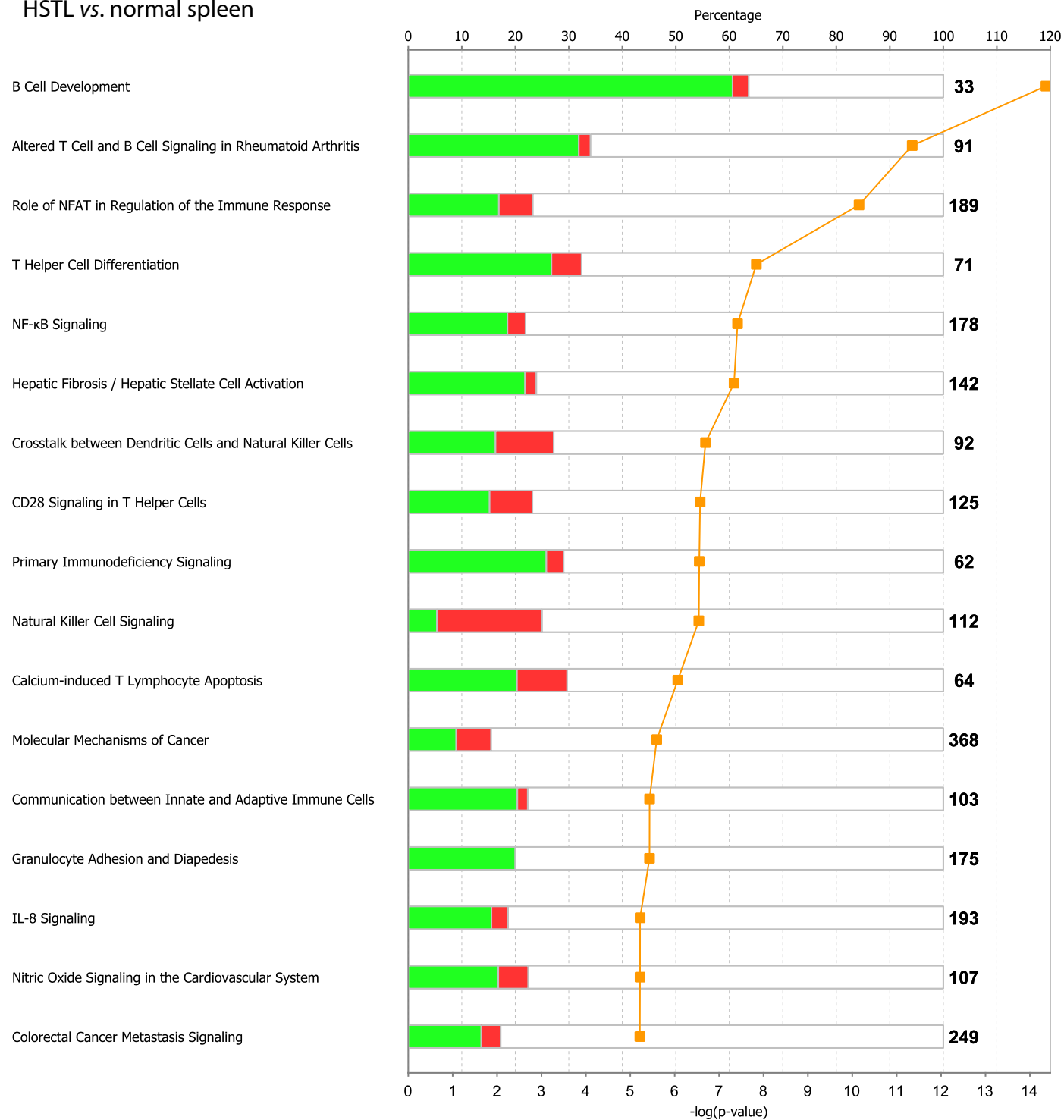

Supplement: Figure S4 — Top dysregulated canonical pathways resulting from individual analysis in IPA. The bold numbers mean the number of molecules involved in a given pathway. The percentage value on the top of the graph means the percentage of dysregulated molecules from the total number of molecules involved in the pathway. Pathways for a given analysis are ranked from higher to lower statistical significance. The statistical significance (p-value) of a given pathway is calculated considering the percentage of dysregulated molecules in the pathways, as well as the fold change of dysregulation. (PDF) [file pone.0102977.s004.pdf]

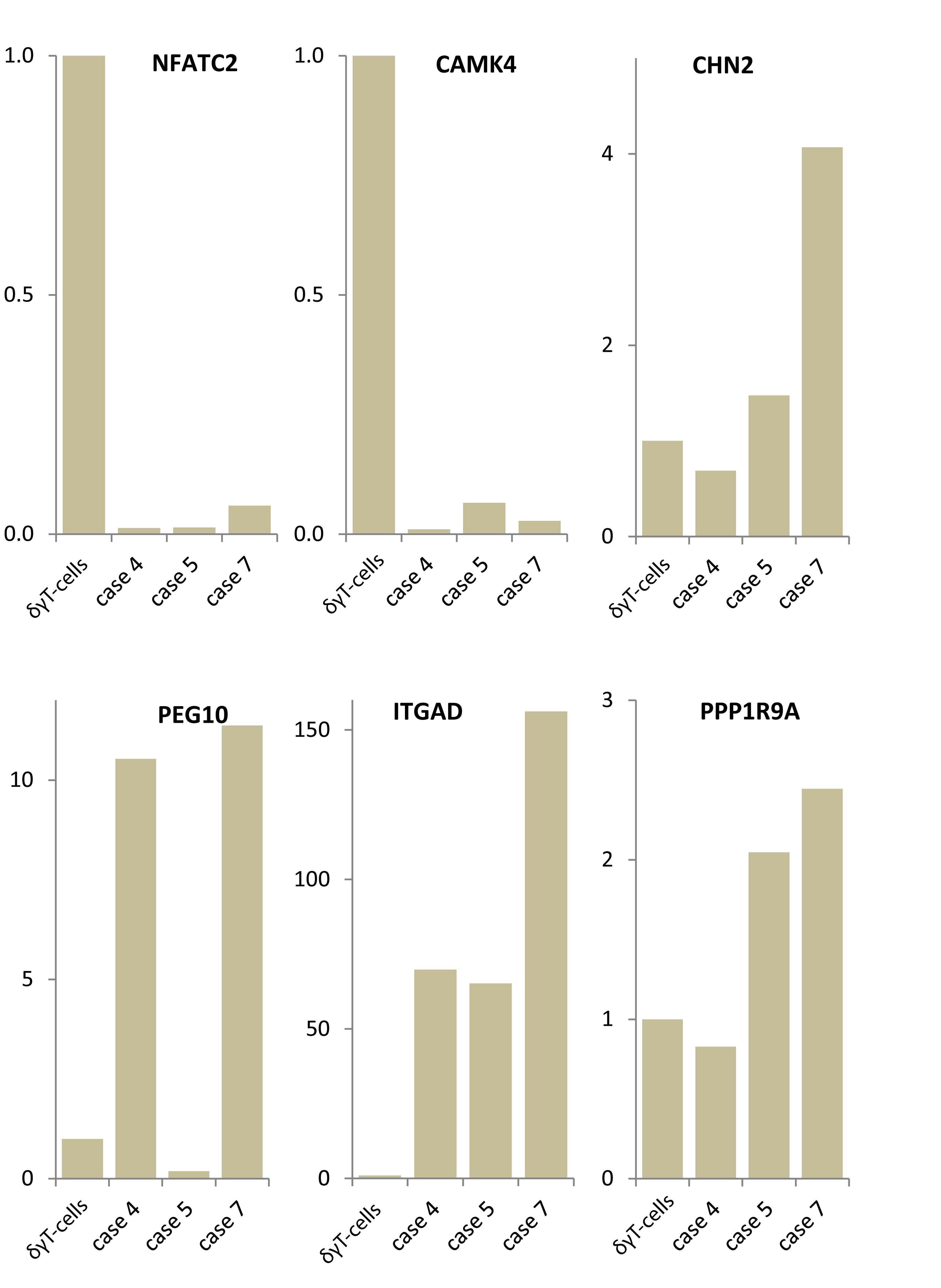

Supplement: Figure S5 — Expression of selected genes analyzed by QRT-PCR. The Y-axis represents the fold change of normalized mRNA expression compared to δγT-cells. (TIF) [file pone.0102977.s005.tif]

**A**

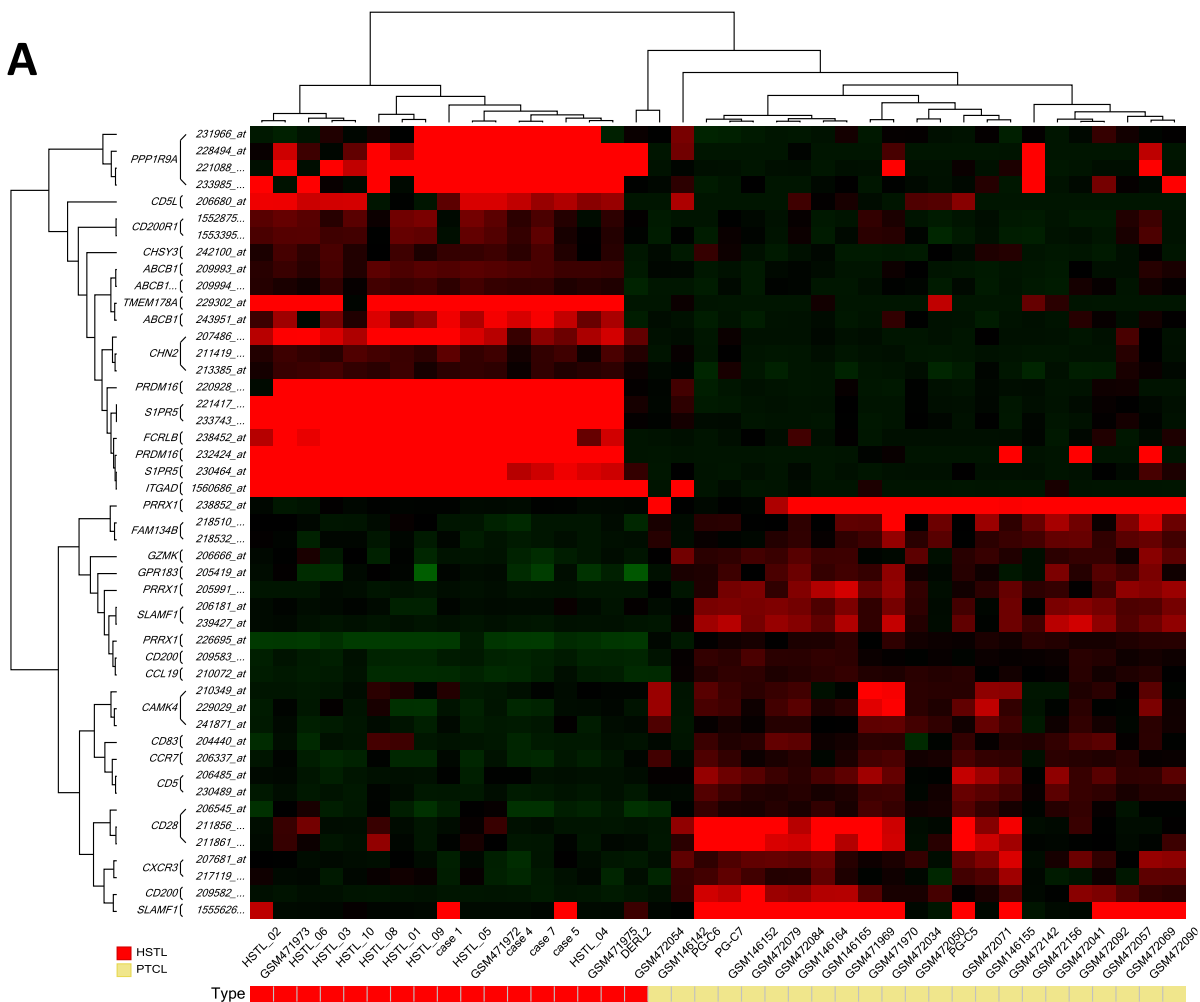

**B**

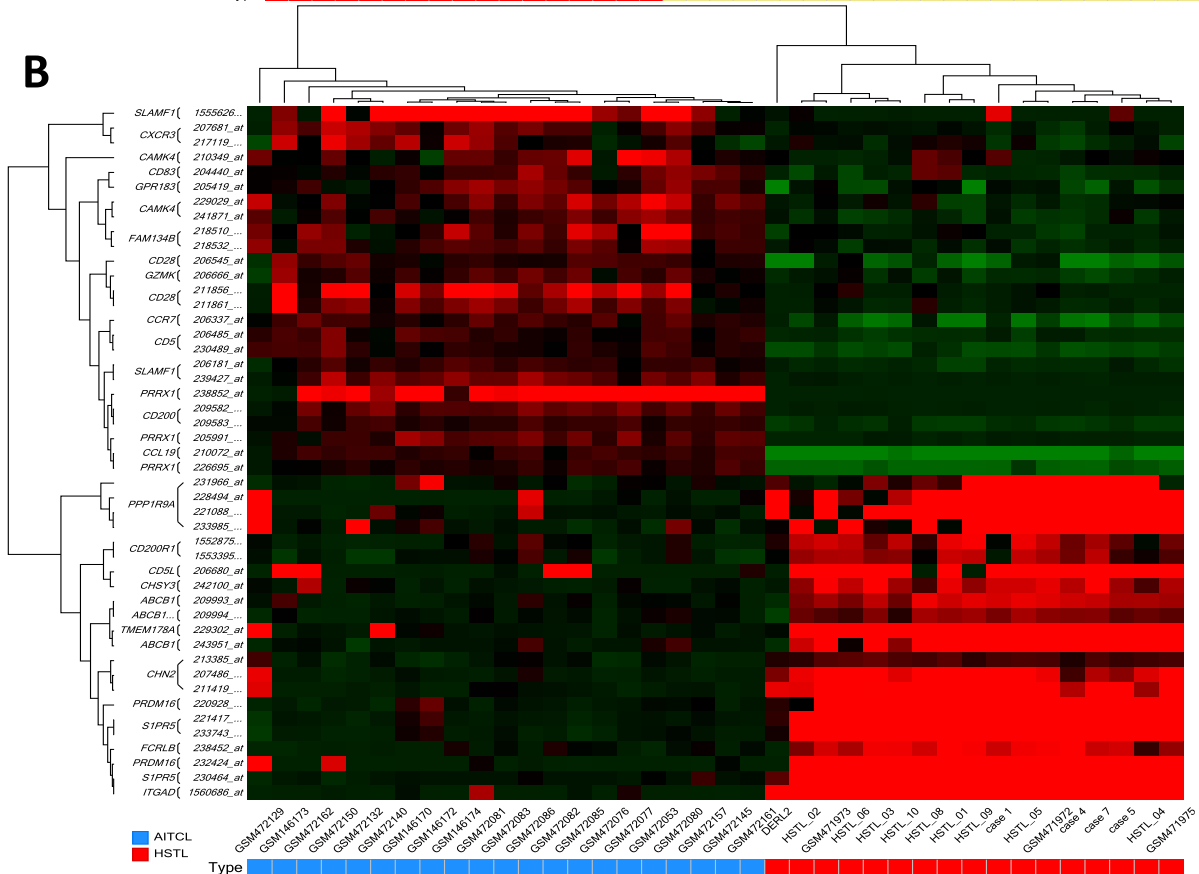

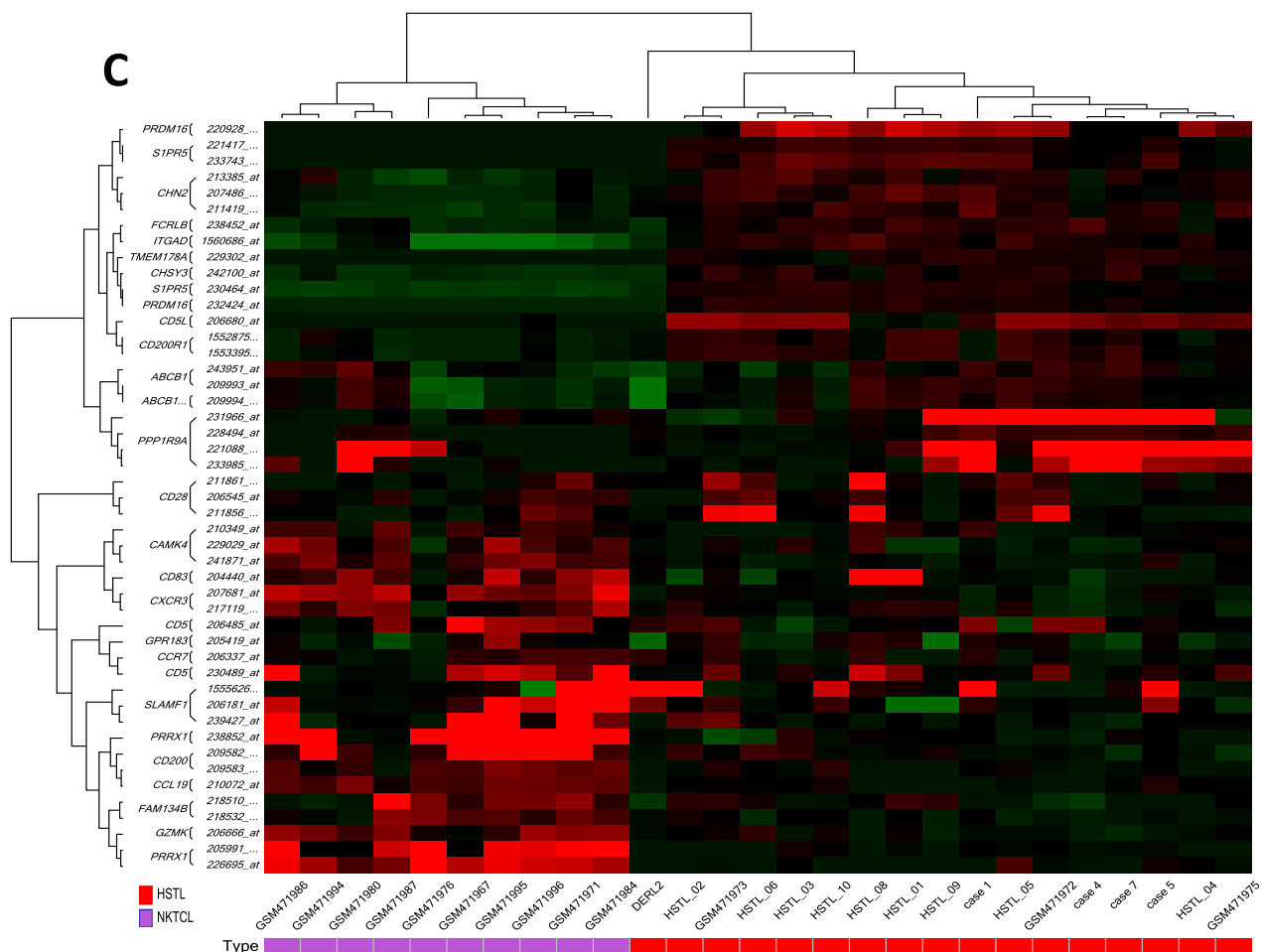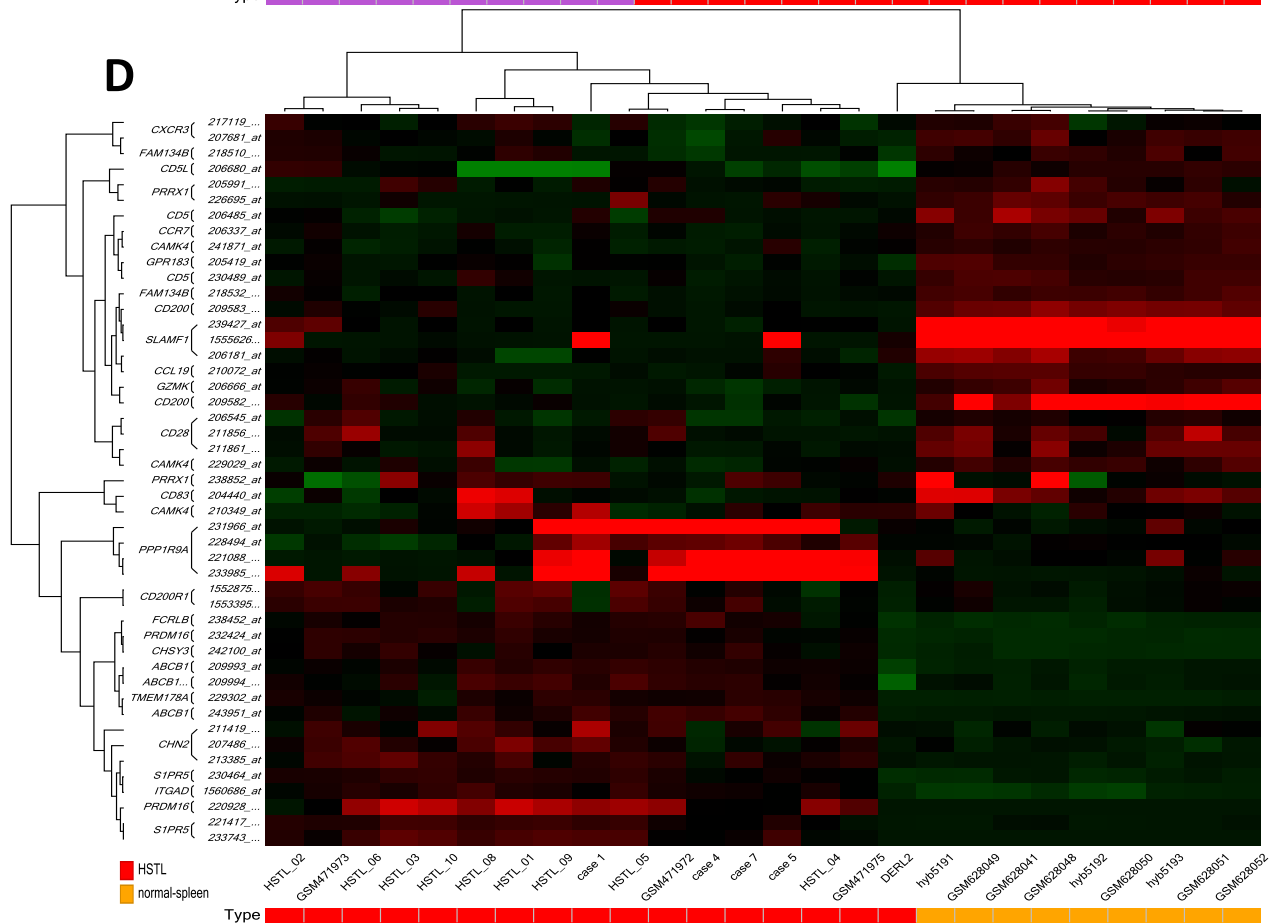

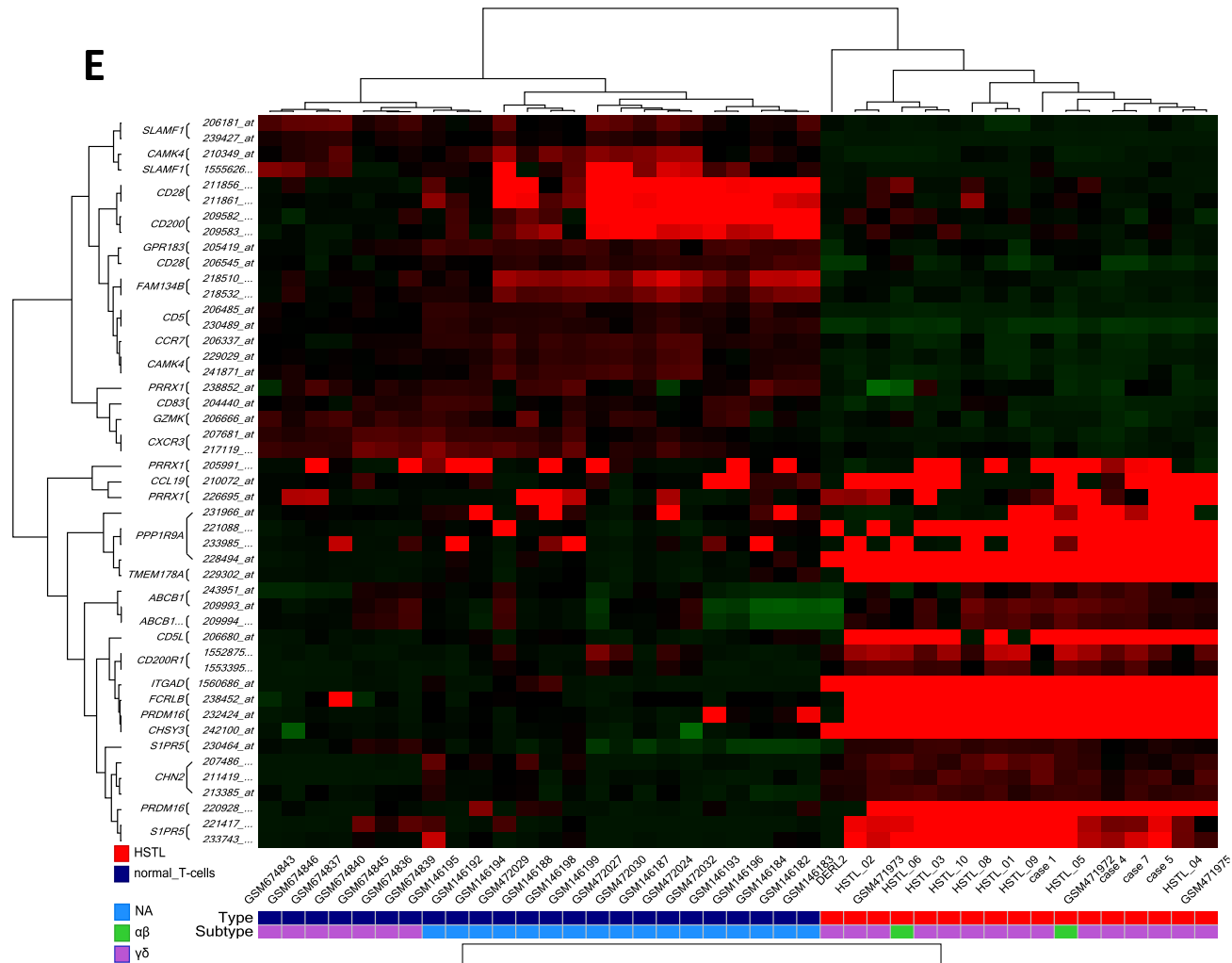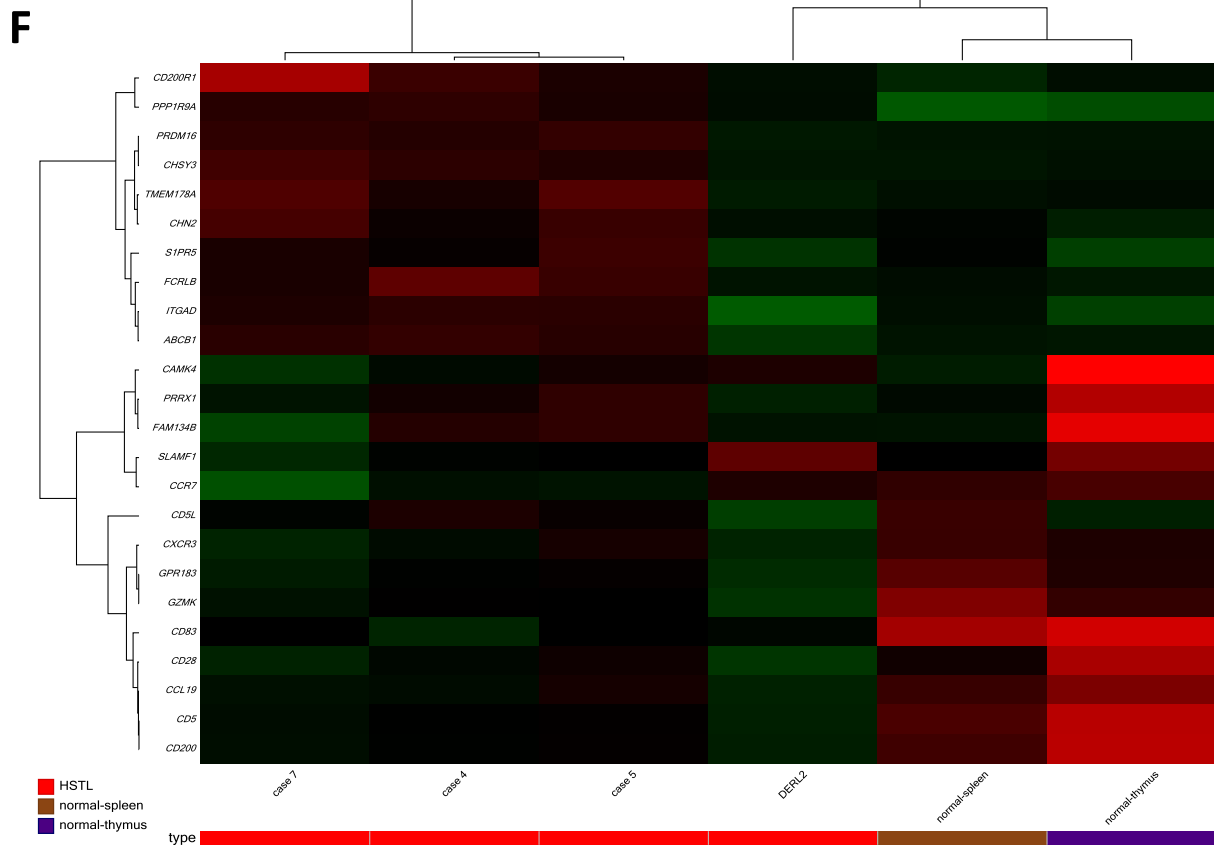

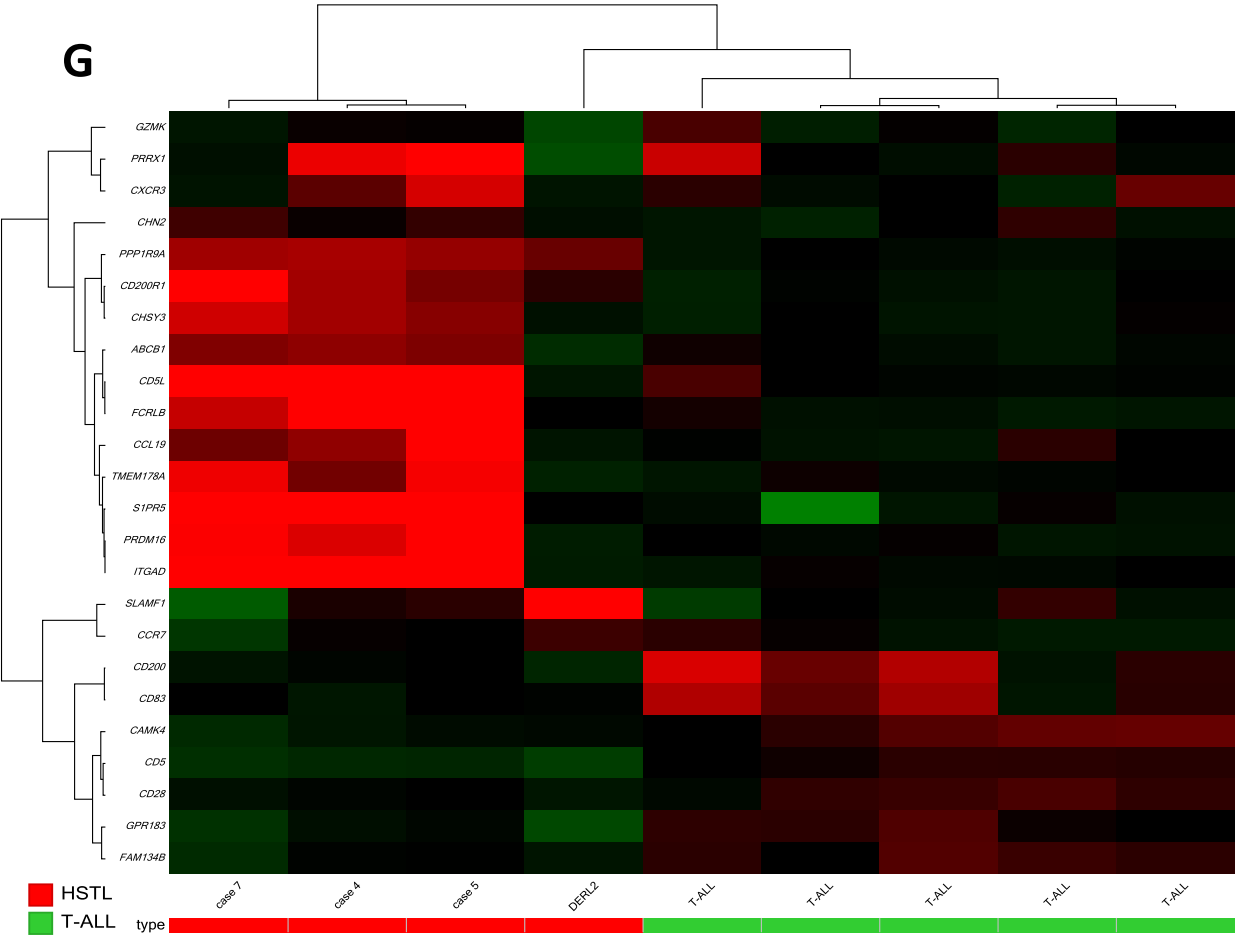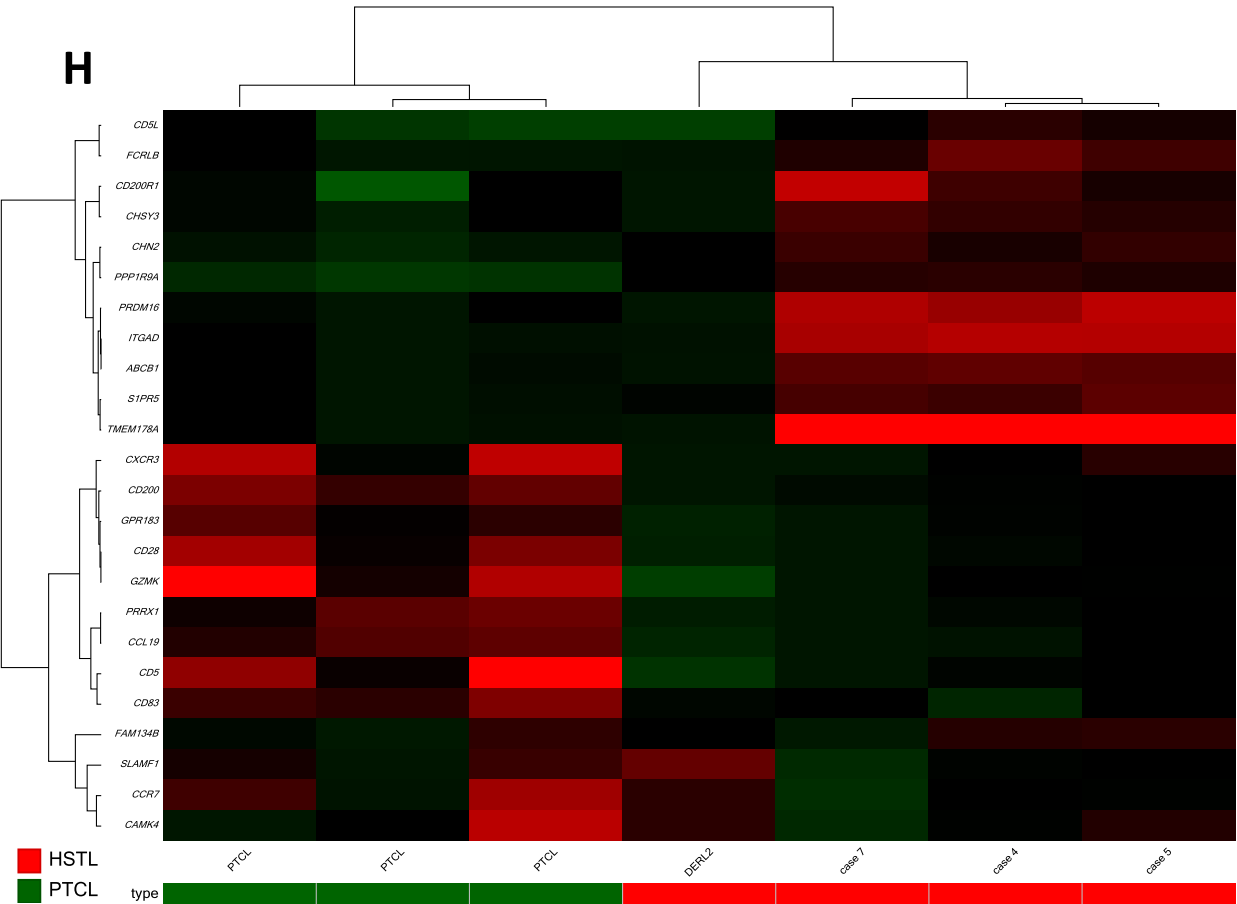

Supplement: Figure S6 — High resolution images of hierarchical clustering using the 24 gene signature for HSTL. The dendograms were generated using the Pearson correlation to calculate the distance and a complete link. The associated heatmap was normalized using a robust center scale. (PDF) [file pone.0102977.s006.pdf]
